# ETHYLENE INSENSITIVE2-like protein mediates submergence and drought responses in *Physcomitrium patens*

**DOI:** 10.1093/plphys/kiaf293

**Published:** 2025-07-02

**Authors:** Md Masudul Karim, Mousona Islam, Marcos Takeshi Miyabe, Yuko Ikeda, Mohammed Arif Sadik Polash, Kanata Hirota, Hidetoshi Sakayama, Yoichi Sakata, Daisuke Takezawa

**Affiliations:** Graduate School of Science and Engineering, Saitama University, Shimo-ohkubo 255, Sakura-ku, Saitama 338-8570, Japan; Department of Crop Botany, Bangladesh Agricultural University, Mymensingh 2202, Bangladesh; Graduate School of Science and Engineering, Saitama University, Shimo-ohkubo 255, Sakura-ku, Saitama 338-8570, Japan; Biological Research Division, Bangladesh Council of Scientific and Industrial Research (BCSIR), Dr. Qudrat-I-Khuda Road, Dhanmondi, Dhaka 1205, Bangladesh; Department of Bioscience, Tokyo University of Agriculture, 1-1-1 Sakuragaoka, Setagaya-ku, Tokyo 156-8502, Japan; Department of Bioscience, Tokyo University of Agriculture, 1-1-1 Sakuragaoka, Setagaya-ku, Tokyo 156-8502, Japan; Graduate School of Science and Engineering, Saitama University, Shimo-ohkubo 255, Sakura-ku, Saitama 338-8570, Japan; Department of Crop Botany, Khulna Agricultural University, Khulna 9202, Bangladesh; Graduate School of Science and Engineering, Saitama University, Shimo-ohkubo 255, Sakura-ku, Saitama 338-8570, Japan; Graduate School of Science, Kobe University, 1-1 Rokkodai-cho, Nada-ku, Kobe 657-8501, Japan; Department of Bioscience, Tokyo University of Agriculture, 1-1-1 Sakuragaoka, Setagaya-ku, Tokyo 156-8502, Japan; Graduate School of Science and Engineering, Saitama University, Shimo-ohkubo 255, Sakura-ku, Saitama 338-8570, Japan

## Abstract

ETHYLENE INSENSITIVE 2 (EIN2) is an Nramp family transmembrane protein recognized as an essential component of ethylene signaling in angiosperms. However, its functions in other plant systems are not fully understood. Here, we demonstrate that *ppein2ab* mutants of the moss *Physcomitrium patens*, in which both *EIN2*-like genes have been disrupted, do not show a typical ethylene-mediated escape response following submergence. Interestingly, *ppein2ab* mutants showed reduced sensitivity to abscisic acid (ABA), a phytohormone that mediates drought stress responses. The *ppein2ab* plants were sensitive to hyperosmosis and freezing stress and exhibited reduced late embryogenesis abundant protein accumulation. Furthermore, *ppein2ab* mutants showed reduced activation of both SNF1-related protein kinase2 (SnRK2), the central activator of ABA and osmostress signaling, and the B3-RAF kinase ARK/PpCTR1L, a positive regulator of SnRK2. These results indicate that EIN2 is a dual function signaling component mediating both submergence and drought signaling in bryophytes. The diminished ABA responses in *ppein2ab* were restored by introduction of Arabidopsis EIN2 and the EIN2 orthologs of the Charophyceaen alga *Chara braunii*, suggesting functional conservation of EIN2 orthologs in Phragmoplastophyta.

## Teaser

A membrane-bound protein designated PpEIN2 of the moss *Physcomitrium patens* participates in the control of stress responses against both waterlogging and water deficit.

## Introduction

Abiotic stress associated with water conditions such as flooding and drought has a great impact on growth and development in plants. While some crop species show diminished yield caused by these water-associated stresses, many plants in nature are equipped with intrinsic mechanisms to sense environmental water levels and exhibit resilience to the stress. Phytohormones play a role in mediating stress responses against waterlogging and water deficit, enabling plants to exert flexible and reversible responses under the condition of changing water availability in the environment. Plant resilience to diverse water stresses is thought to be brought about by sensing and signaling molecules and signal cross-talk between phytohormones, although the underlying mechanisms are largely unknown.

Ethylene is well known for its role in regulation of plant growth, senescence, and fruit ripening in angiosperms, but it also is known as a mediator of submergence responses in vegetative tissues. Submergence results in rapid accumulation of ethylene in tissues, which triggers escape responses such as the formation of elongated stems in rice (Hattori et al. 2009). Submergence also induces ethylene-mediated aerenchyma formation to facilitate gas exchange in adventitious roots in monocots (Yamauchi et al. 2014, 2016). Submergence response mediated by ethylene also occurs in nonangiosperm plants such as ferns and liverworts (Musgrave and Walters 1974; Stange and Osborne 1988). In the moss *Physcomitrium patens*, submergence causes the formation of short-branched protonemata with enhanced gametophore production, mediated by ETHYLENE TRIPLE RESPONSE1 (ETR1)-like histidine kinase (ETR-HK) (Yasumura et al. 2012).

In the signaling pathway proposed in Arabidopsis, ethylene is perceived by histidine-kinase receptors, ETR1, ETR2, ETHYLENE RESPONSE SENSOR 1 (ERS1), ERS2 and ETHYLENE-INSENSITIVE 4 (EIN4) localized in the endoplasmic reticulum (ER) membrane. These receptors play a role in the regulation of the B3-Raf kinase CONSTITUTIVE TRIPLE RESPONSE 1 (CTR1) for downstream signaling (Shakeel et al. 2015; Zhao et al. 2021). In the absence of ethylene, CTR1 is active and phosphorylates ER-membrane-bound ETHYLENE-INSENSITIVE 2 (EIN2), preventing it from proteolytic cleavage. The binding of ethylene to the receptors causes inhibition of CTR1, leading to a cleavage of EIN2 and localization of the EIN2 C-terminal end domain (CEND) to the nucleus. CEND stabilizes the transcription factors ETHYLENE-INSENSITIVE 3 (EIN3) and EIN3-like (EIL), leading to activation of the downstream transcription factors that stimulate ethylene responses (Wen et al. 2012; Zhang et al. 2017).

While ethylene mediates the submergence response, abscisic acid (ABA) is known to mediate stress caused by drought in plants (Métraux and Kende 1983; Cao et al. 2013). Evidence of antagonistic actions of ethylene and ABA by a signal crosstalk between these phytohormones has been obtained in studies mainly using Arabidopsis mutants with altered sensitivity to ethylene. For instance, seed germination of ethylene-insensitive *etr1-1* and *ein2-1* mutants was hypersensitive to ABA, and *ctr1-1* with a constitutive ethylene response was insensitive to ABA (Beaudoin et al. 2000; Ghassemian et al. 2000). Such antagonistic interactions between ethylene and ABA are attributed to reciprocal regulation of their endogenous levels (Cheng et al. 2009; Li and Huang 2011), but other mechanisms have also been proposed (Yu et al. 2019; Guo et al. 2023). Interestingly, it has also been reported that root growth is synergistically inhibited by ethylene and ABA. Roots of both *etr1-1* and *ein2* mutants showed insensitivity to ABA in Arabidopsis (Beaudoin et al. 2000; Ghassemian et al. 2000), in which the ethylene signaling cascade but not ethylene biosynthesis is required for inhibition of root growth by ABA. Synergistic interaction in roots and antagonistic interaction in coleoptiles between ABA and ethylene have also been reported in rice (Ma et al. 2014), but whether the mechanism is different from that of Arabidopsis is not known.

In aforementioned reports showing possible interactions between ethylene and ABA, however, there is little information about the role of SNF1-related protein kinase 2 (SnRK2), which is known to be the primary activator of ABA and osmostress signaling (Fujii and Zhu 2009; Umezawa et al. 2009). SnRK2s comprise part of the core signaling module of ABA signaling with PYRABACTIN RESISTANCE1 (PYR)/PYR1-LIKE (PYL)/REGULATORY COMPONENTS OF ABA RECEPTORS (PYR/PYL/RCAR) and Group A protein phosphatases 2C (PP2CAs) (Ma et al. 2009; Park et al. 2009). SnRK2 activity is suppressed by PP2CAs in the absence of ABA, but binding of ABA to the PYR/PYL/RCAR receptor causes inhibition of PP2CAs, thus activating SnRK2. Activated SnRK2 then phosphorylates and activates downstream factors including ion channels and transcription factors (Cutler et al. 2010).

In bryophytes, analysis of mutants in *P. patens* has revealed a possible mechanism for the integrated regulation of flooding and drought responses by ER-localized ETR-HK and the B3-RAF kinase ARK, the latter also known as *P. patens* CTR1-like (PpCTR1L) (Yasumura et al. 2015; Toriyama et al. 2022). ARK/PpCTR1L activates SnRK2 by phosphorylating 2 serine residues in the activation loop, and ETR-HK is required for activation of ARK/PpCTR1L (Saruhashi et al. 2015; Toriyama et al. 2022). Disruption of either *ARK*/*PpCTR1L* or 4 *ETR*-*HK* genes (*PpHK5*/*PpHK13*/*PpHK20*/*PpHK24*) in *P. patens* abolishes ABA- and osmostress-induced activation of SnRK2, accompanied by drastic reductions in *LATE EMBRYOGENESIS ABUNDANT* (*LEA*)-like gene expression and osmostress tolerance. These disruptants (*ark/ppctr1l* and *pphk5*/*13*/*20*/*24*) also showed a constitutive escape response with or without submergence, indicating that ETR-HK and ARK/PpCTR1L also regulate the ethylene-mediated submergence response (Toriyama et al. 2022).

In this study, we investigated the role of orthologs of EIN2, a factor that is thought to work downstream of ETR-HK, in water-stress responses in *P. patens*. Analysis of genome-editing lines indicated that the null mutants of the *P. patens EIN2* orthologs (*ppein2ab*) show reduced sensitivity to exogenous ABA, with reduced activity of SnRK2 and ARK/PpCTR1L, and consequent reduced osmostress tolerance. The mutants also lacked a submergence response in growth and gene expression, indicating that *P. patens* EIN2, with ETR-HK and ARK/PpCTR1L, is a member of the dual-function signaling components for drought and submergence responses.

## Results

### Role of EIN2 orthologs in the osmostress response in *P. patens*

**Figure 1.**
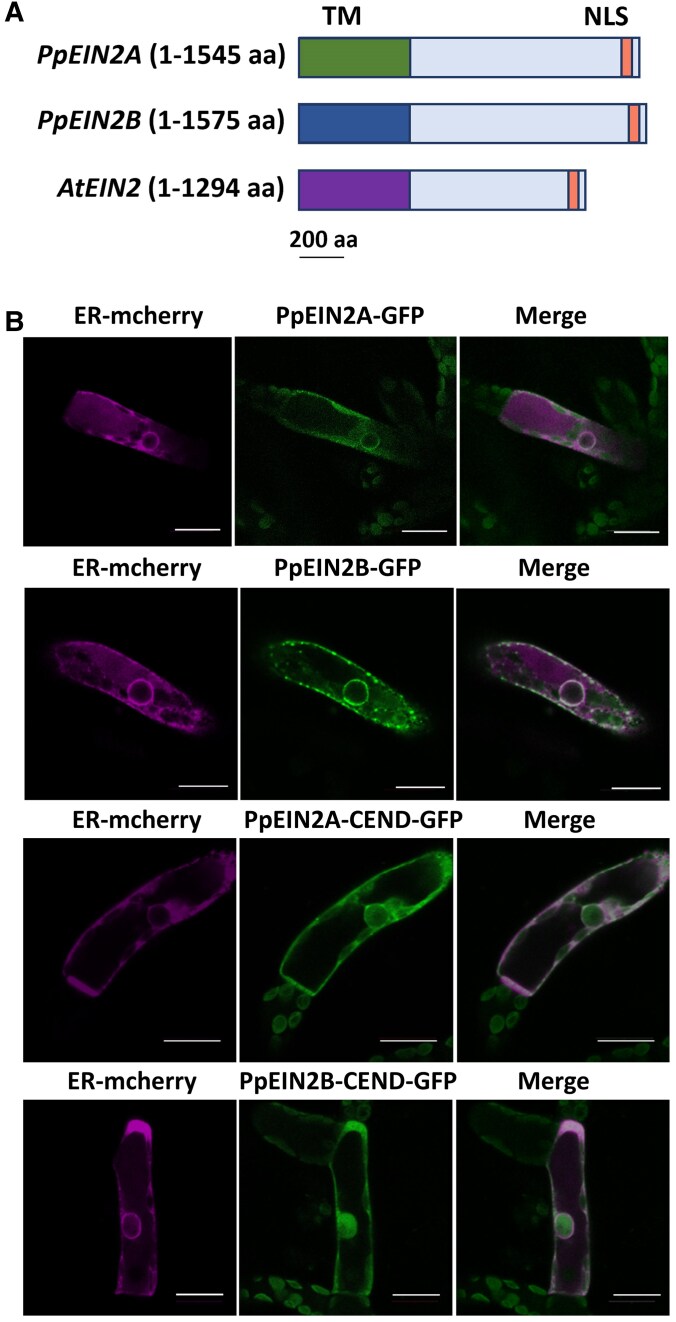
Schematic structures and localization of EIN2-like proteins of *Physcomitrium patens*. **A)** Polypeptide structures of PpEIN2A, PpEIN2B, and AtEIN2. The TM and the putative NLS are denoted. **B)** Localization of the GFP-fusion proteins of PpEIN2A, PpEIN2B, and the C-terminus region (CEND). The fluorescent protein mCherry with the ER-localization signal (ER-mCherry) was used as a control for the ER-localization. Expression of all the fluorescent proteins was driven by the *Actin* promoter. The scale bar indicates 50 μm.

There are 2 *EIN2*-like genes in *P. patens*, *PpEIN2A* (*Pp3c16_16830V3.1*) and *PpEIN2B* (*Pp3c27_2050V3.1*), encoding polypeptides of 1,545 and 1,575 amino acids, respectively. They are structurally similar to Arabidopsis *EIN2* (*AT5G03280*), each containing both a conserved N-terminal transmembrane (TM) domain and a CEND region containing a putative nuclear localization signal (NLS) (Fig. 1A).

To analyze the cellular localization of PpEIN2A and PpEIN2B, GFP-fusion constructs with the full-length coding regions were introduced into protonema cells of *P. patens* by particle bombardment. GFP fluorescence signals of both PpEIN2A and PpEIN2B were largely overlapped with ER-mCherry, the ER localization marker (Fig. 1B, upper panels). We also analyzed the localization of CEND constructs for each EIN2 ortholog without the TM domain, and we found that for both PpEIN2A-CEND and PpEIN2B-CEND, fluorescence was localized in both the ER and nucleus (Fig. 1B, lower panels).

To determine the role of the EIN2 orthologs in *P. patens*, we generated genome-editing lines by targeting the 19-nucleotide sequence ts1 common in both *PpEIN2A* and *PpEIN2B* in the CEND region. Furthermore, single disruptants for each gene and additional double mutants, *ppein2ab_38* and *ppein2ab_39*, were generated by targeting the sequences ts2 and ts3 in the transmembrane domains (TMs) (Supplementary Fig. S1, A and B).

**Figure 2.**
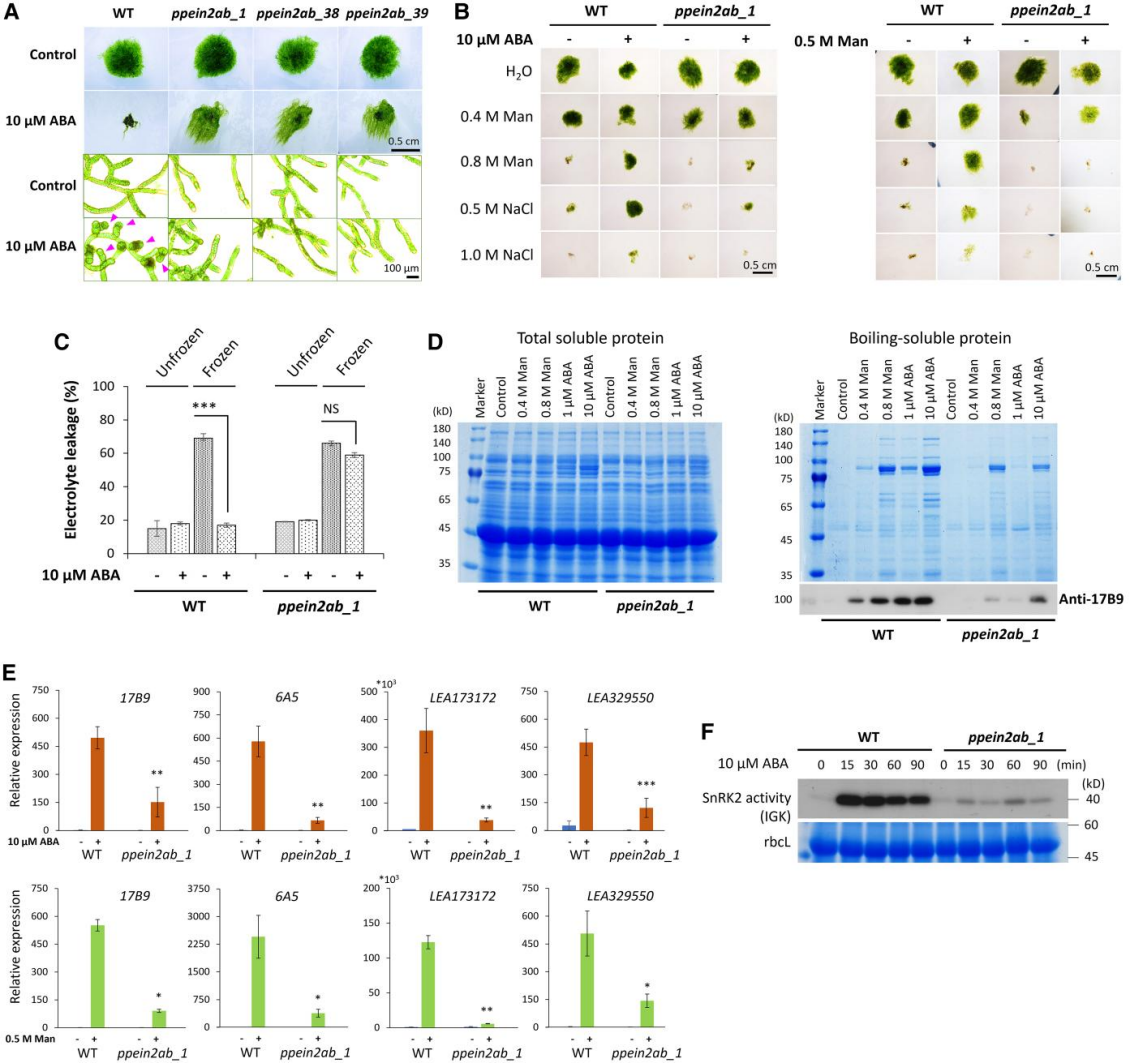
ABA and abiotic stress responses in the *ppein2ab* lines. **A)** Effect of ABA on growth of wild type (WT) and 3 *ppein2ab* lines. Plants were grown in medium with and without 10 μM ABA for 2 weeks. The arrowheads indicate the brood cells in WT, which are not found in the *ppein2ab* lines. The scale bar of 0.5 cm is applicable for all the colonies and that of 100 µm is applicable for all the cell images. **B)** Tests for osmostress tolerance with 10 μM ABA and 0.5 m mannitol (Man) pretreatments. Cultured protonemata were pretreated with 10 μM ABA or 0.5 m mannitol for 1 day. Both control and pretreated protonema were exposed to different concentrations of Man and NaCl for 15 min as osmostress, and then cultured in normal growth media for 10 days to determine survival. The scale bar (0.5 cm) is applicable for all the colonies. **C)** Tests for freezing tolerance. The protonemata were pretreated with or without 10 μM ABA for 1 day and frozen to −4°C. After thawing, percentages of electrolyte leakage were determined. Treatment means were adjusted in one-way ANOVA with the standard error (SE) of treatment means (*n* = 4, ***P < 0.001). NS denotes nonsignificant (*P* > 0.05). **D)** SDS-PAGE analysis of total soluble proteins and boiling-soluble proteins. Cultured protonemata were treated with different concentrations of Man and ABA for 1 day and total soluble proteins were extracted. The protein samples were boiled for 1 min and after centrifugation, the supernatant was used as the boiling-soluble protein fraction. The boiling-soluble proteins were used for the Coomassie Brilliant Blue (CBB) staining and the immunoblot analysis with the anti-17B9 antibody. **E)** Reverse transcription-quantitative PCR (RT-qPCR) analysis for the *LEA*-like genes. Primers for *17B9* (*Pp3c23_13700V3.1*), *6A5* (*Pp3c4_880V3.1*), *LEA173172* (*Pp3c13_20930V3.1*), and *LEA329550* (*Pp3c3_29550V3.1*) were used. Cultured protonemata were treated with 10 μM ABA or 0.5 m Man for 6 h and the extracted total RNA was used for the SYBR Green-based RT-qPCR analysis using *Actin* as an internal control. Error bars indicate standard error of treatment means (*n* = 3), and the statistical means are adjusted in Student's *t*-test (*P < 0.05, **P < 0.01, ***P < 0.001). **F)** SnRK2 activity was detected by the in-gel kinase assay (IGK) using histone-IIIS as a substrate. The CBB staining shows the large subunit of Rubisco (rbcL) as the loading control.

To determine whether the *ppein2ab* lines show altered ABA sensitivity, protonemata of the *ppein2ab* lines were grown on a medium containing 10 µM ABA. Growth of WT protonemata was severely inhibited by ABA with formation of propagules called brood cells for the vegetative reproduction. In contrast, the growth of *ppein2ab* protonemata showed reduced ABA sensitivity, and typical brood cells were not observed (Fig. 2A, Supplementary Fig. S2). In contrast, the ABA response in the *ppein2a* and *ppein2b* single disruptants was similar to that of WT (Supplementary Fig. S3A).

We previously showed that WT protonemata acquire osmostress tolerance upon pretreatment with ABA or a mild osmostress (Takezawa et al. 2015). Thus, we analyzed changes in osmostress tolerance in the representative *ppein2ab_1* line. Osmostress tests indicated that the WT protonemata acquired tolerance to 0.8 m mannitol and 0.5 m NaCl after pretreatment with 10 µM ABA, while the *ppein2ab_1* protonemata did not (Fig. 2B, left). Furthermore, mild osmostress treatment with 0.5 m mannitol induced tolerance to 0.8 m mannitol in WT but not in *ppein2ab_1* (Fig. 2B, right). We also analyzed freezing tolerance of the cells, by which levels of the tolerance to dehydration stress can be estimated quantitatively, by measurement of electrolyte leakage (Nagao et al. 2005). Pretreatment with 10 µM ABA reduced electrolyte leakage after freezing and thawing in WT, indicating enhancement of tolerance to freeze-induced dehydration. In contrast, electrolyte leakage was not significantly reduced by ABA pretreatment in *ppein2ab_1* (Fig. 2C). These results indicate that the *EIN2*-like genes are necessary for both osmostress and freezing tolerance. Tolerance to these stresses in *ppein2a* and *ppein2b* single disruptants was similar to that in WT (Supplementary Fig. S3, B and C).

It has been shown that ABA and osmostress induce accumulation of LEA-like boiling-soluble proteins that protect cells from dehydration damage (Knight et al. 1995). Analysis of total and boiling-soluble protein fractions of protonemata by SDS-polyacrylamide gel electrophoreiss followed by Coomassie Brilliant Blue staining indicated that both ABA and mannitol treatments induce accumulation of boiling-soluble proteins in WT. However, the levels of accumulation were reduced in *ppein2ab_1*, especially when lower concentrations of ABA and mannitol were used (Fig. 2D). Results of immunoblot analysis using an antibody against 17B9, one of the LEA-like proteins, indicated reduced accumulation of 17B9 in response to ABA and mannitol treatment in *ppein2ab_1* (Fig. 2D). We analyzed expression of 4 representative *LEA*-like genes, *17B9* (*Pp3c23_13700V3.1*), *6A5* (*Pp3c4_880V3.1*), *LEA173172* (*Pp3c13_20930V3.1*), and *LEA329550* (*Pp3c3_29550V3.1*), before and after treatment with ABA and mannitol by the reverse transcription quantitative PCR (RT-qPCR) analysis. We found that expression of all 4 genes was significantly reduced in *ppein2ab_1* in comparison with that in WT (Fig. 2E). Furthermore, we analyzed activation of SnRK2 upon ABA treatment by in-gel kinase assays using radioactive ATP and histone IIIS as substrates. In the WT protonemata, ABA treatment induced rapid activation of SnRK2 within 15 min and the activation lasted until 90 min. In contrast, SnRK2 activity was only slightly induced by ABA treatment and the level of activation remained low during the treatment in *ppein2ab_1* (Fig. 2F).

**Figure 3.**
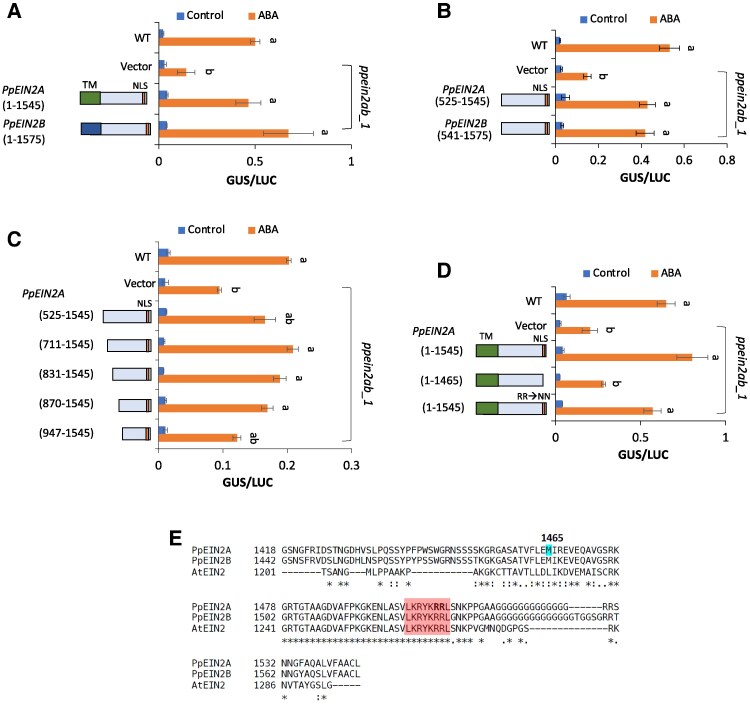
Determination of PpEIN2 domains responsible for the ABA response. **A–D)** Reporter assays of *ppein2ab_1* to examine restoration of ABA response. Cultured protonemata were bombarded with the ABA-inducible *Em* promoter fused with *beta-glucuronidase* (*proEm-GUS*) as the reporter, the rice *Ubiquitin* promoter fused with *luciferase* (*proUbi-LUC*) as the reference and various effector constructs of cDNAs. After bombardment, the protonemata were cultured for 1 day with or without ABA, and *GUS* and *LUC* activity was determined to evaluate ABA response. The results were compared with those of WT protonemata bombarded without the effector constructs. **A)** Comparison between full length cDNAs of *PpEIN2A* and *PpEIN2B*. **B)** Effect of *CEND* of *PpEIN2A* and *PpEIN2B*. **C)** N-terminal deletions in *CEND* of *PpEIN2A*. **D)** C-terminal deletions and substitution of 2 Arg with Asn in the putative NLS. **E)** Comparison of amino acids near the critical deletion endpoint among the EIN2 orthologs. An asterisk (*) indicates identical residues, a colon (:) indicates conserved substitutions, and a dot (.) indicates semiconserved substitutions. The position of the amino acid 1,465 shown in **D)** is highlighted. The putative NLS sequences (LKRYKRRL) are marked with 2 bolded Arg residues altered to Asn in **D)**. For all assays, comparison is made using one-way ANOVA and the error bar represents SE (*n* = 3). Different letters represent the statistical difference (*P* < 0.05).

Extents of ABA-induced gene expression can be examined by transient reporter assays using ABA-inducible promoters (Marella et al. 2006). For detailed analyses of the functions of PpEIN2, reporter assays were conducted using WT and *ppein2ab_1* protonema cells. The cells were bombarded with the *beta-glucuronidase* (*GUS*) gene fused to the ABA-inducible *Em* promoter (*proEm-GUS*) and the *luciferase* (*LUC*) gene fused to the *Ubiquitin* promoter (*proUbi-LUC*), with or without *PpEIN2* cDNA fused to the *Actin* promoter. The cells were then cultured with or without ABA for 1 day and used for GUS and LUC assays. Bombardment with *proEm*-*GUS* and *proUbi*-*LUC* revealed that *ppein2ab_1* showed a lower GUS/LUC ratio indicating reduced ABA response than did WT, but cobombardment with *PpEIN2A* or *PpEIN2B* cDNA restored the ABA response to a level similar to that of WT (Fig. 3A). This result, with the results of stress tolerance in single disruptants (Supplementary Fig. S3, B and C), indicates that *PpEIN2A* and *PpEIN2B* are functionally redundant for the ABA response. We also tested *PpEIN2A* and *PpEIN2B* constructs without the TM domain. We found that these constructs also restored the ABA response in *ppein2ab_1*, indicating that the *CEND* region of both is sufficient for the ABA response in this assay (Fig. 3B).

To determine the amino acid regions critical for the ABA response, we carried out deletion analysis of *PpEIN2A* by reporter assays. We found that deletion of amino acids from the N-terminus to 1,018 abolishes its function (Supplementary Fig. S4A). Further deletion analysis revealed that deletions up to amino acid 869 from the N-terminus do not affect the restoration of ABA response in *ppein2ab*, but a deletion to 946 reduced the ABA response (Fig. 3C). On the other hand, deletions from the C-terminus revealed that the construct without the C-terminal 80 amino acids containing putative NLS failed to restore the ABA response in *ppein2ab_1* (Fig. 3D, Supplementary S4B). In an attempt to determine the role of the putative NLS, we replaced 2 arginine residues (Arg1505 and Arg1506) (Fig. 3E) with asparagine residues, which should disrupt the NLS function, and introduced the construct into *ppein2ab* protonemata. However, this mutant restored ABA response in a manner similar to that of full-length *PpEIN2A*, indicating that NLS itself may not be critical for the ABA response (Fig. 3D).

### Introduction of Arabidopsis EIN2 and an algal EIN2 ortholog into *ppein2ab*

**Figure 4.**
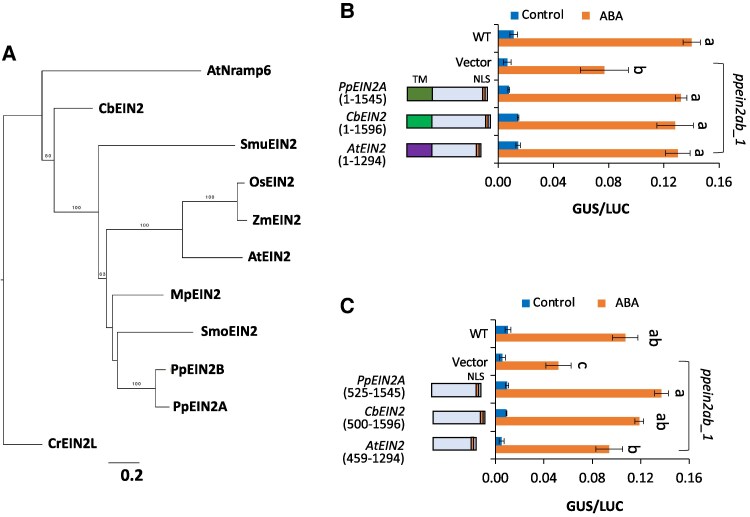
Possible role of EIN2 orthologs in abscisic acid (ABA) responses. **A)** Phylogenetic tree of *EIN2*-like genes constructed by RAxML-NG with the LG + I + G4 + F model. Amino acid sequences of representative *EIN2*-like genes were aligned, and 730 sites in conserved regions were used for the analysis. Bootstrap analysis was performed with 1,000 replicates, and the percentage values (≥50%) are indicated on each branch. The horizontal branch lengths are proportional to the estimated number of substitutions per site. The horizontal branch lengths are proportional to the estimated number of substitutions per site. AtEIN2, *Arabidopsis thaliana* AT5G03280; OsEIN2, *Oryza sativa* Os07g06130; ZmEIN2, *Zea mays* Zm00001d039341; SmoEIN2, *Selaginella moellendorffii* 447098; CbEIN2, *Chara braunii* CHBRA353g00160; SmuEIN2, *Spirogloea muscicola* SM000027S09592; MpEIN2, *Marchantia polymorpha* Mp1g18880; AtNramp6, *A*. *thaliana* Nramp6 (AT1G15960). *Chlamydomonas reinhardtii* EIN2-like (CrEIN2L, Cre07.g315200.t1) was used as an outgroup. **B, C)** Reporter assays of *ppein2ab_1* to examine restoration of ABA response. Cultured protonemata were bombarded with the ABA-inducible *Em* promoter fused with *beta-glucuronidase* (*proEm-GUS*) as the reporter, the rice *Ubiquitin* promoter fused with *luciferase* (*proUbi-LUC*) as the reference and various effector constructs of cDNAs. After bombardment, the protonemata were cultured for 1 day with or without ABA, and *GUS* and *LUC* activity was determined to evaluate ABA response. The results were compared with those of WT protonemata bombarded without the effector constructs. **B)** Comparison among *PpEIN2A*, *Arabidopsis EIN2* (*AtEIN2*), and the *Chara braunii EIN2* ortholog *CbEIN2*. **C)** Comparison among the *CEND* regions of *PpEIN2A*, *AtEIN2*, and *CbEIN2*. For all assays, comparison is made using one-way ANOVA and the error bar represents Se (*n* = 3). Different letters represent the statistical difference (*P* < 0.05).

Phylogenetic analysis indicated that *EIN2*-like genes are conserved not only in embryophytes but also in some streptophyte algae such as *Chara braunii* and *Spirogloea muscicola* (Fig. 4A). We tested the functions of Arabidopsis *EIN2* (*AtEIN2*) and the *EIN2* ortholog of *C. braunii* (*CbEIN2*) in the ABA response to explore functional conservation in streptophytes. We chose *C. braunii* as a source, because Charophyceae to which it belongs is a clade sister to all other groups within Phragmoplastophyta, and *C. braunii* has a gene encoding an intact EIN2 ortholog with both the TM domain and the CEND region (Supplementary Fig. S5). Reporter assays using the *ppein2ab* protonemata revealed that introduction of the cDNAs of both *AtEIN2* and *CbEIN2* restored the ABA response to a level similar to that in WT (Fig. 4B). Furthermore, constructs without the TM domain of these cDNAs also restored the ABA response (Fig. 4C).

### Interactions of PpEIN2 with ETR-HK and ARK/PpCTR1L

**Figure 5.**
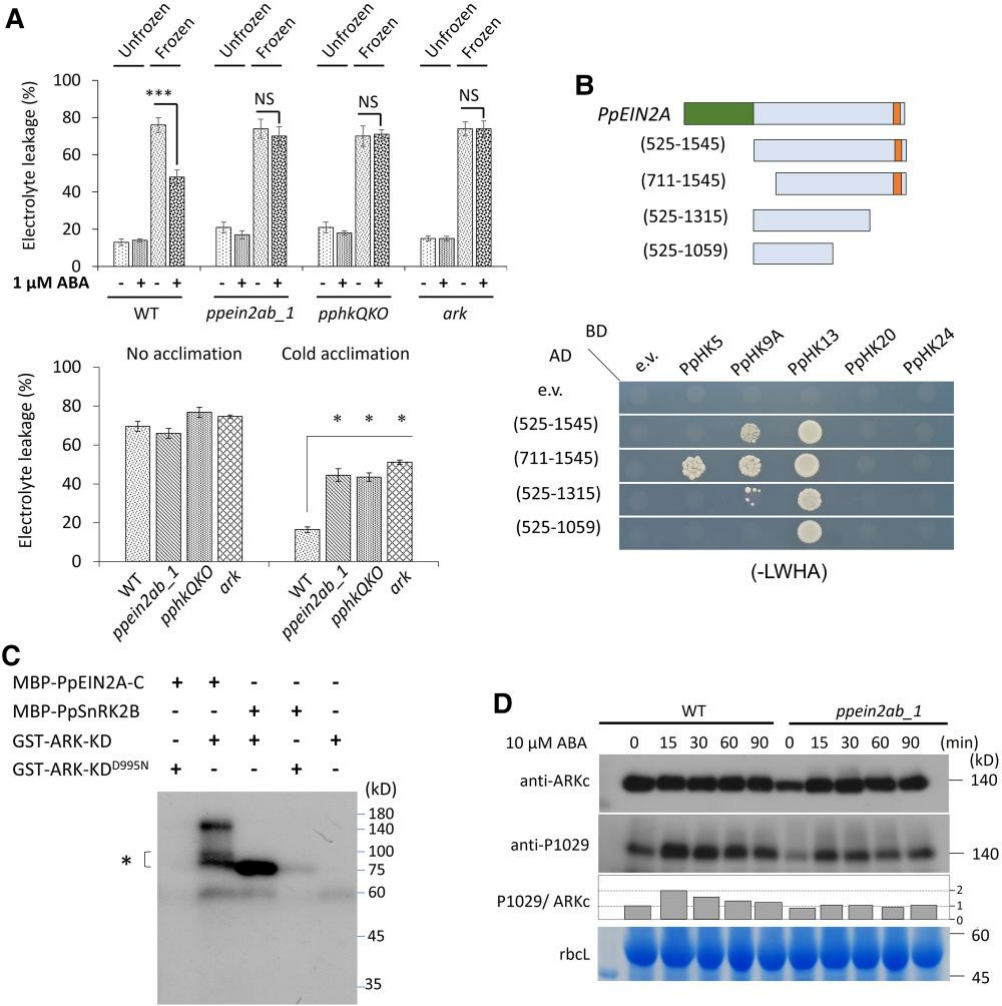
Interaction analysis of PpEIN2 with PpHK and ARK/PpCTR1L. **A)** Tests for freezing tolerance in the *P. patens* wild type (WT) and mutant lines. Protonemata of WT, *ppein2ab_1*, *pphkQKO*, and *ark/ppctr1 l* (*ark*) were pretreated with ABA (upper) or cold (lower), and either kept unfrozen or frozen to −3 °C. Electrolyte leakage was measured after thawing to determine freezing injury. Comparison is made against cold acclimated WT protonema separately using the Student's *t*-test (*n* = 3, *P < 0.05, ***P < 0.001). **B)** Yeast two-hybrid assays for examination of the interaction between PpEIN2A and PpHK isoforms. Haploid yeasts carrying the GAL4 activation domain (AD)-fused PpEIN2A constructs and the GAL4 binding domain (BD)-fused PpHK5, PpHK9A, PpHK13, PpHK20, or PpHK24 constructs (Toriyama et al. 2022) were mated and grown on the synthetic defined (SD) medium lacking Leu/Trp/His/Ade (-LWHA). Amino acid regions in PpEIN2A used for the assays are shown above the photos of yeast colonies. **C)** In vitro protein kinase assays of recombinant proteins. Maltose binding protein (MBP)-fused PpEIN2A-CEND (PpEIN2A-C) and PpSnRK2B were phosphorylated with the glutathione S-transferase (GST)-fused ARK/PpCTR1L kinase domain (ARK-KD) with or without the D995N mutation, which disrupts kinase activity (Islam et al. 2021). Proteins expressed in *Escherichia coli* and purified by affinity chromatography were reacted with gamma-^32^P-ATP at 30 ˚C for 15 min and electrophoresed. Radioactive signals were detected by autoradiography. The asterisk indicates degradation products of MBP-PpEIN2A-C. **D)** Immunoblot analysis using anti-ARK C-terminus (ARKc) and anti-Ser1029-phosphorylated ARK (P1029) antibodies. Sizes of the molecular markers are shown in kilodalton (kD). The histograms indicate the ratio of Ser1029-phosphorylated ARK to ARKc, assuming the value of 0 h-treated WT as 1, analyzed by Gel Analyzer version 23.1.1. Coomassie Brilliant Blue-stained large subunit of Rubisco (rbcL) bands are shown as the loading controls.

Previous studies have shown that Arabidopsis EIN2 directly interacts with ETR-HK and CTR1 (Bisson and Groth 2015; Ju et al. 2012). In *P. patens*, both ETR-HK and ARK/PpCTR1L are required for the ABA response and stress tolerance (Saruhashi et al. 2015; Toriyama et al. 2022), but how ETR-HK and ARK/PpCTR1L affect or are affected by PpEIN2 remains unclear. Tests for freezing tolerance in response to ABA and cold indicate that the tolerance in *ppein2ab_1*, *ark*/*ppctr1l*, and *pphkQKO* (*pphk5/13/20/24*) is reduced to similar levels in comparison with that in WT (Fig. 5A, Supplementary Fig. S6). To determine the interaction of PpEIN2 with ETR-HK and ARK/PpCTR1L, yeast two-hybrid assays were carried out using *CEND* fused to the activation domain or the binding domain of the yeast *GAL4* sequences. We found that CEND of PpEIN2A (525–1,545) interacts with 2 ETR-HKs, PpHK9A, and PpHK13, and that PpEIN2B (541–1,575) interacts with PpHK13 (Fig. 5B;  Supplementary Fig. S7A). Deletion analysis of PpEIN2A revealed that PpEIN2A (711–1,545) interacts with PpHK5 in addition to PpHK9A and PpHK13, and PpEIN2A (525–1,059) interacts with PpHK13 only (Fig. 5B). These results indicate that PpEIN2A can interact with more than 1 member of ETR-HKs. In contrast, there was no interaction between PpEIN2A and ARK/PpCTR1L (Supplementary Fig. S7B).

Next, we explored the possibility of phosphorylation of PpEIN2A by ARK/PpCTR1L. We used the *Escherichia coli*-expressed GST-fusion protein of the ARK/PpCTR1L kinase domain (GST-ARK-KD), which phosphorylates and activates one of SnRK2s designated PpSnRK2B (Saruhashi et al. 2015), for in vitro kinase assays with the maltose-binding protein-fused PpEIN2A-CEND (MBP-PpEIN2A-C). We found that GST-ARK-KD phosphorylates MBP-PpEIN2A-C, while GST-ARK-KD with a D995N mutation, which disrupts the kinase activity (Islam et al. 2021), did not (Fig. 5C).

We previously reported that ABA treatment induces autophosphorylation at Ser1029 of ARK/PpCTR1L in the activation loop of the kinase domain, which is critical for the ABA response in *P. patens* (Islam et al. 2021). We carried out immunoblot analysis of WT and *ppein2ab_1* protonemata using the same proteins as those used for the in-gel kinase assays for which results are shown in Fig. 2F. The proteins were reacted with the anti-ARKc antibody that recognizes the C-terminal 15 amino acids of ARK/PpCTR1L for estimation of protein accumulation (Saruhashi et al. 2015) and the anti-P1029 antibody that recognizes the Ser1029-phosphorylation to estimate levels of ARK activation. We found that the level of Ser1029 phosphorylation relative to ARK was increased in WT, but the activation levels were lower in *ppein2ab_1* (Fig. 5D). These results suggest that reduction in SnRK2 activity is in parallel with the reduced phosphorylation of ARK/PpCTR1L.

### Submergence response in *ppein2ab*

**Figure 6.**
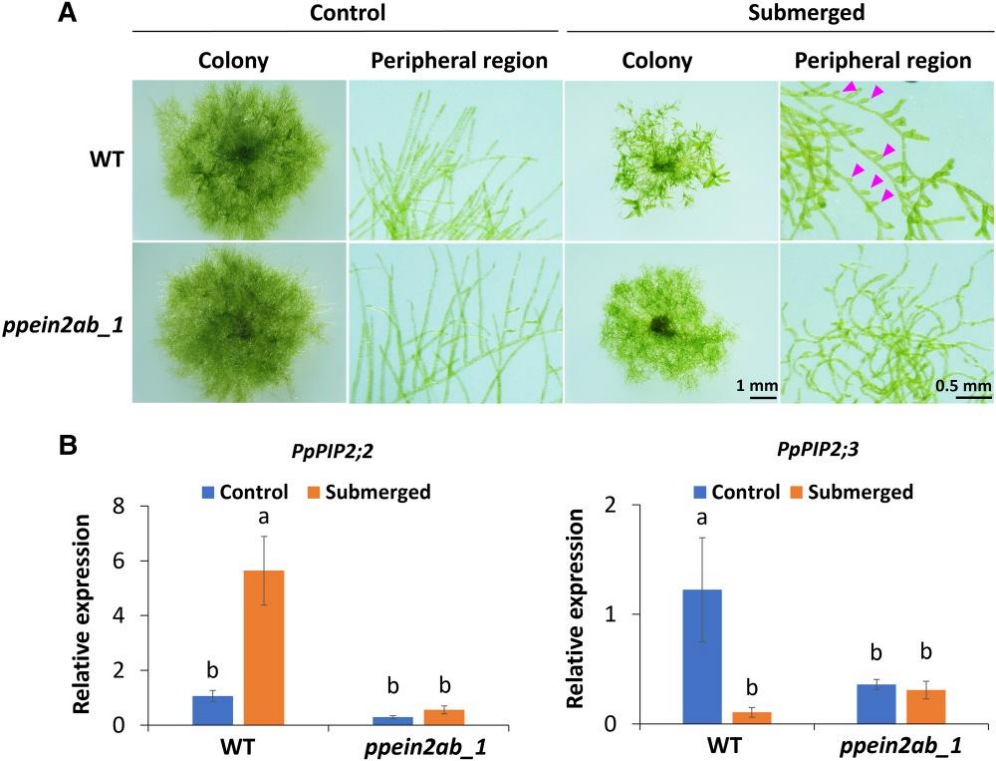
Role of EIN2-like proteins of *Physcomitrium patens* in the submergence response. **A)** Growth response under submergence in wild type (WT) and *ppein2ab_1*. Cultured protonemata were spotted on agar media and cultured for 2 days. Then, 30 mL of sterile water was gently added to each plate and further cultured for 3 weeks under continuous light. The arrowheads indicate short branches of protonemata. The scale bar indicates 1 mm (colony) and 0.5 mm (peripheral region), and is applicable for all other images. **B)** Reverse transcription-quantitative PCR (RT-qPCR) analysis for submergence-responsive gene expression. RNA extracted from the protonemata was reverse-transcribed and analyzed by the SYBR Green-based qPCR method with *Tubulin* as the internal control. Standard error (SE) of means (*n* = 4) is indicated. One-way analysis of variance (ANOVA) was used to compare the means among different groups. Different letters represent the statistical difference (*P* < 0.01).

We analyzed whether the *ppein2ab* lines show altered submergence responses in addition to the reduced osmostress responses. Wild-type plants submerged in water for 2 weeks showed a typical escape response with short-branched protonemata as reported previously (Yasumura et al. 2012; Toriyama et al. 2022). In contrast, such a response was not observed in *ppein2ab_1* by the submergence treatment (Fig. 6A). The submergence response of the *ppein2a* and *ppein2b* single disruptants and the complementation line was similar to that of WT (Supplementary Figs. S8 and S9). We analyzed the expression of the submergence-upregulated *PpPIP2;2* gene (*Pp3c3_31900V3.1*) and the submergence-downregulated *PpPIP2;3* gene (*Pp3c4_510V3.1*), which are also upregulated and downregulated by ethylene, respectively (Yasumura et al. 2012). The results of the RT-qPCR analysis indicated that the expression levels of both genes were lower than that of WT and were not changed by the submergence treatment (Fig. 6B). These results indicate that *P. patens* EIN2 is critical for the submergence responses in protonemata.

### Role of *EIN3*-like genes in ABA, osmostress, and submergence responses

**Figure 7.**
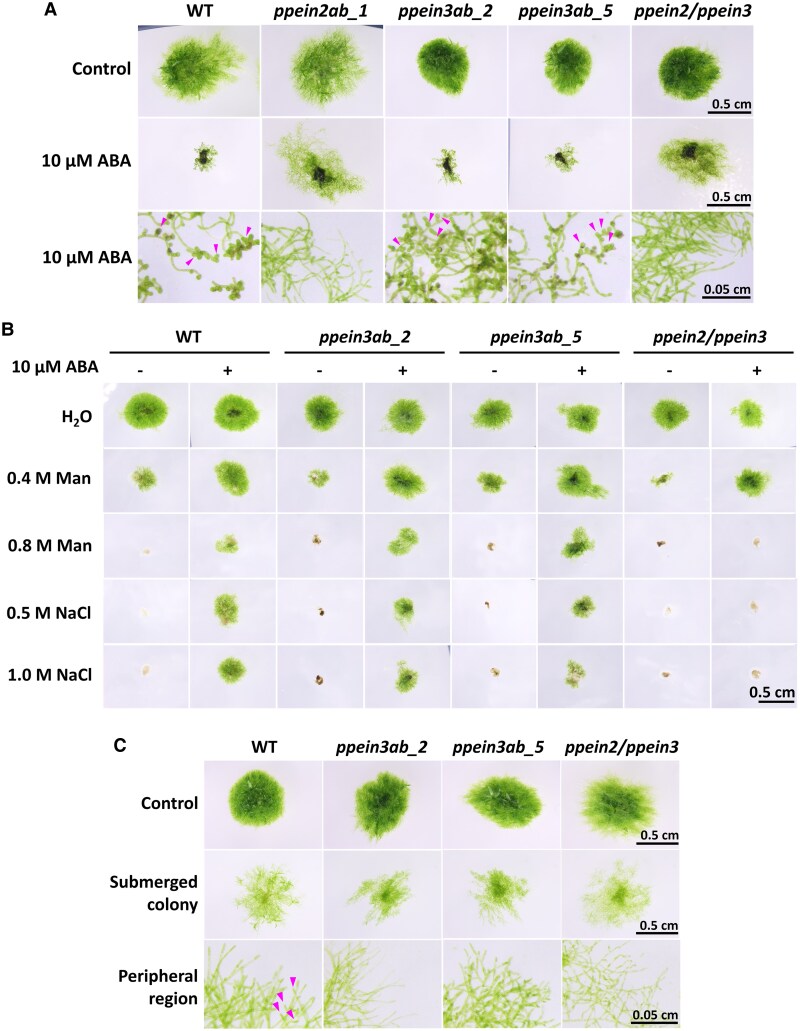
Role of *EIN3*-like genes of *P. patens* in abscisic acid (ABA), osmostress and submergence responses. **A)** Growth response analysis with the application of 10 µM ABA. *ppein3ab_2 and ppein3ab_5* are the double knockout lines of 2 *EIN3*-like genes, *PpEIN3A* and *PpEIN3B,* and *ppein2/ppein3* is the quadruple knockout line of *PpEIN2A*, *PpEIN2B*, *PpEIN3A*, and *PpEIN3B*. Plants were grown on medium with and without the 10 μM ABA for 2 weeks. The arrowheads indicate brood cells. **B)** Osmostress tolerance tests. Cultured protonemata were pretreated with or without 10 μM ABA for 1 day, exposed to the indicated concentrations of mannitol and NaCl for 15 min as osmostress, and then cultured in normal growth media for 10 days to determine survival. **C)** Protonemata spotted on agar media were cultured for 2 days, to which 30 mL of sterile water was gently added for the submergence treatment. The arrowheads indicate short branches of protonemata. The plates were then cultured for 3 weeks under continuous light. The scale bar indicates 0.5 cm for colonies and 0.05 cm for cells of the peripheral region.

In the canonical ethylene response pathway, the transcriptional regulators EIN3 and EIL are activated by EIN2-CEND translocated to the nucleus (An et al. 2010). There are 2 genes encoding EIN3/EIL-like proteins in the *P. patens* genome (Mao et al. 2022), and we designated these genes as *PpEIN3A* (*Pp3c7_9970V3.1*) and *PpEIN3B* (*Pp3c11_15260V3.1*). We conducted genome editing to target both of these genes and obtained 2 independent lines, *ppein3ab_2* and *ppein3ab_5*, and also the *ppein2*/*ppein3* quadruple mutant (Supplementary Fig. S10). Analysis of ABA and osmostress experiments indicated that the both *ppein3ab* mutants showed the ABA responses similar to those in WT, with respect to growth inhibition, brood cell formation, and stress tolerance (Fig. 7, A and B; Supplementary Fig. S11). In contrast, the *ppein2*/*ppein3* quadruple mutant showed phenotypes similar to those of the *ppein2ab* mutants. On the other hand, the *ppein3ab* mutants showed submergence responses similar to those of *ppein2ab* (Fig. 7C; Supplementary Fig. S12). These results indicate that the *EIN3* orthologs of *P. patens* function downstream of *PpEIN2* in the ethylene-signaling pathway for the submergence response, while *PpEIN2* also functions in the ABA-mediated osmostress response.

## Discussion

### PpEIN2 is a member of the signal complex for submergence and osmostress responses

**Figure 8.**
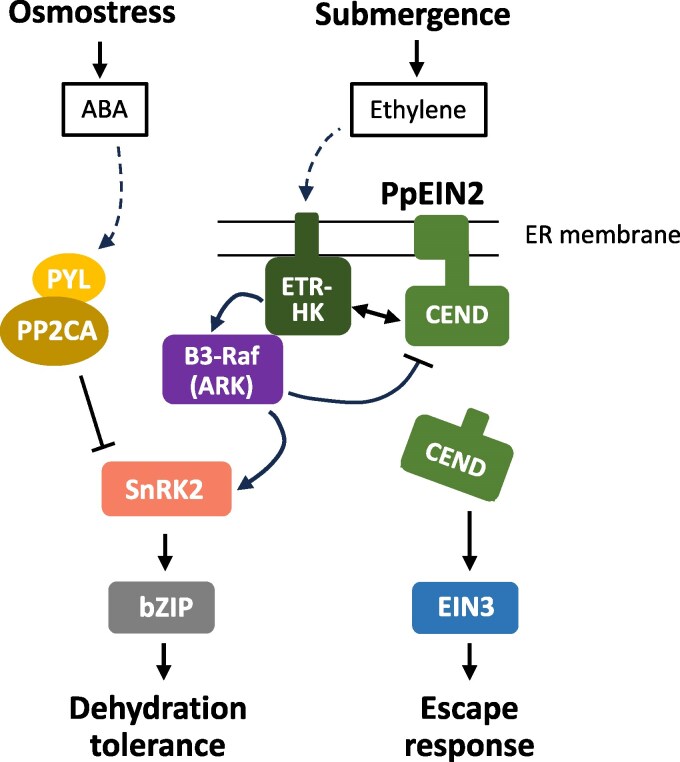
Proposed working model showing the roles of PpEIN2 in the regulation of osmostress and submergence responses in *Physcomitrium patens*. PpEIN2 mediates submergence signaling through the regulation of the EIN3 ortholog. PpEIN2 also affects the osmostress signaling pathway by regulating B3-Raf (ARK/PpCTR1L) and SnRK2 through the interaction with ETR-HK indicated by a double arrow. The solid lines with pointed ends indicate positive influence and the lines with blunted ends indicate inhibition. Binding of phytohormones to the receptors are indicated by dashed lines with pointed ends.

In this study, we showed possible roles of EIN2 orthologs in submergence and ABA/osmostress responses in *P*. *patens* using null *ppein2ab* mutant lines. Orthologs of EIN2 are present in seed plants, lycophytes, and bryophytes, but physiological studies of EIN2 have been focused on that in angiosperms. It was only recently reported that disruption of *PpEIN2B* in *P. patens* causes a loss of ethylene-induced gametophore production (Wang et al. 2024). Our results showing the loss of escape responses and gene expression under the condition of submergence in null *ppein2ab* mutants indicate that *PpEIN2* plays a role in the submergence response (Fig. 6), consistent with the result of a previous study showing constitutive escape response phenotypes in protonemata of the *pphkQKO* and *ark*/*ppctr1l* mutants (Toriyama et al. 2022). In addition, impaired ABA responses and stress tolerances in *ppein2ab* in association with drastic reductions in SnRK2 activity (Fig. 2, A–F) indicate that PpEIN2 is a signaling component for the ABA-mediated osmostress responses as well. Results showing interaction between PpEIN2A and 3 ETR-HKs and reduced autophosphorylation of ARK/PpCTR1L, which also interacts with ETR-HKs, in *ppein2ab_1* (Fig. 5, B and D) suggest that PpEIN2 can form a signaling complex with ETR-HKs and ARK/PpCTR1L on the ER membrane (Fig. 8). Considering that both ETR-HK and ARK/PpCTR1L are necessary components for activation of SnRK2 (Saruhashi et al. 2015; Toriyama et al. 2022), the results obtained in this study indicate that ETR-HK-ARK/PpCTR1L-PpEIN2 consists of a dual-function signal complex for drought and submergence responses mediated by ABA and ethylene, respectively.

### Roles of CEND in ABA signaling

Several previous studies indicate that CEND of EIN2 plays a key role in ethylene signal trafficking from the ER to the nucleus. Constitutive expression of AtEIN2 CEND partially activates ethylene responses (Alonso et al. 1999), and removal of the NLS from CEND eliminates localization to the nucleus and abolishes ethylene signaling (Wen et al. 2012). CEND translocated to the nucleus stabilizes EIN3/EIL1, which activates genes encoding ethylene response factor family transcription factors (Chao et al. 1997; Solano et al. 1998). On the other hand, there are also reports showing a role of CEND outside the nucleus. CEND can inhibit degradation of EIN3/EIL by repressing translation of EIN3-Binding F-box 1 (EBF1) and EBF2 in the cytoplasmic P-body (Gagne et al. 2004; Li et al. 2015; Merchante et al. 2015). It has also been reported that NLS in CEND plays a cryptic role involving the ETR1–EIN2 interaction and that removal of NLS strongly reduced the affinity for ETR1 (Bisson and Groth 2015). Although there are several reports in which the possible role of EIN2 in the regulation of ABA response is described (Beaudoin et al. 2000; Ghassemian et al. 2000; Cheng et al. 2009; Ma et al. 2014; Thole et al. 2014), the role of CEND in the ABA response was examined only by Guo et al. (2023), who reported that interaction of EIN2-CEND with HOOKLESS 1 in the nucleus might be a mechanism for the repression of ABA responses in *ein2*.

In this study, we showed that PpEIN2A-CEND, especially the stretch of highly conserved C-terminal 80 amino acids, is important for the ABA response in *P. patens* (Fig. 3D). It appears that the C-terminal region is also important for the PpEIN2B function (Supplementary Fig. S13). Whether the putative NLS (LKRYKRRL) in this region is critical for the ABA response is not clear, since replacement of 2 Arg residues with Asn did not affect the recovery of the ABA response (Fig. 3D). In our observations, localization of GFP fused with the full-length PpEIN2A or PpEIN2B to the nucleus was not observed with or without ABA treatment (Fig. 1B; Supplementary Fig. S14). Although we cannot exclude the possibility of translocation of a small portion of CEND-GFP to the nucleus upon ABA treatment, our results indicate that nuclear localization of CEND may not be necessary for the ABA response. This possibility was supported by experiments with the disruptants of the *EIN3* orthologs (Fig. 7), indicating that the PpEIN2-mediated ABA response does not require activation of PpEIN3 in the nucleus. Furthermore, expression of *PpEIN2A_CEND* in the ABA-insensitive *ark*/*ppctr1l* and *pphk*QKO lines did not restore ABA response, suggesting that the function of PpEIN2 in the ABA response is dependent on ETR-HK and ARK/PpCTR1L localized on the ER membrane (Supplementary Fig. S15).

It is possible that a lack of PpEIN2 causes destabilization of ETR-HK during ABA treatment, as transient assays of protonemata indicated that the introduced PpHK5-LUC is more stable in WT than in the *ppein2ab* mutant (Supplementary Fig. S16). If so, the C-terminal amino acid region in CEND might play a role in the regulation of ETR-HK through a direct interaction on the ER membrane (Bisson and Groth 2015). Functions of PpEIN2 might be regulated by phosphorylation by ARK/CTR1L (Fig. 5C), although how this phosphorylation affects the ABA response is presently unknown. The phosphorylation of PpEIN2 might provide a feedback mechanism for the regulation of ARK/PpCTR1L activity, if CEND of PpEIN2 is important for stabilization of ETR-HK. Experiments are being conducted to determine the phosphorylation sites by ARK/PpCTR1L in the PpEIN2 polypeptide and reporter assays are being conducted with phosphomimic and nonphosphorylatable mutants of PpEIN2.

### Is the role of EIN2 orthologs in osmostress response common in streptophytes?

Our results provide important evolutionary insights into the function of EIN2 in streptophytes. Restoration by AtEIN2 of the ABA response in *ppein2ab* (Fig. 4, B and C) indicates that EIN2 might also play a role in ABA response in angiosperms by functioning with ETR-HK and B3-Raf. In Arabidopsis, the *ein2* mutant shows either insensitivity or hypersensitivity to ABA, depending on tissues (Beaudoin et al. 2000; Ma et al. 2014). This difference might be due to tissue-specific regulation of transcription factors such as ABI5 and ABI3 (Guo et al. 2023), but the difference might also be due to the diversity of CTR1-related B3-Raf kinases, some of which might participate in the regulation of both ABA and ethylene responses in a tissue-specific manner. While ARK/PpCTR1L is the only B3-Raf in *P. patens*, Arabidopsis has 6 B3-Rafs such as CTR1 that participates in ethylene signaling, EDR1 involved in plant immunity (Tang et al. 2005) and 3 AtARKs responsible for activation of SnRK2 in osmostress responses (Katsuta et al. 2020; Takahashi et al. 2020). It has been shown that the ABA-resistant phenotype of *abi1-1* seeds is enhanced in *ctr1* (Beaudoin et al. 2000). Furthermore, AtARK3 (At4g24480) interacts with ETR1 and ERS1, and its mutant *atark3* showed reduced sensitivity to the ethylene precursor 1-aminocyclopropane-1-carboxylic acid (Yasumura et al. 2015). How the B3-Raf kinases other than CTR1 in Arabidopsis regulate EIN2 function during ABA and ethylene responses is presently unknown.

Recent genome analyses revealed that genes for ethylene-signaling factors had first appeared in streptophyte algae (Nishiyama et al. 2018; Cheng et al. 2019), but, interestingly, the distribution of EIN2 orthologs varies depending on the alga classes. For instance, both *Mesotaenium endlicherianum* (Zygnematophyceae) and *Klebsormidium nitens* (Klebsormidiophyceae) appear to lack EIN2 orthologs but have intact ETR-HK and EIN3 orthologs. The Zygnematophyceae alga *Spirogyra pratensis* exhibits a cell elongation in response to ethylene, and its orthologs of ethylene-signaling factors homologous to ETR-HK and EIN3 partially rescued mutants of Arabidopsis (Ju et al. 2015). However, the ortholog of EIN2 in *Spirogyra* apparently lacks the TM domain and the function of the polypeptide has not been determined. Our results showing restoration of ABA response in *ppein2ab* by introduction of the EIN2 ortholog of *Chara braunii* indicate the possible role of *EIN2*-like genes in the osmostress response in Charophyceae, which belongs to Phragmoplastophyta. *C*. *braunii* also has orthologs of ETR-HK, CTR1 (B3-Raf), and EIN3, indicating that the ethylene response pathway is also conserved in Charophyceae. Considering the functional conservation of SnRK2 in streptophytes (Shinozawa et al. 2019), the establishment of crosstalk between submergence and osmostress facilitated by the ETR-HK–B3-Raf–EIN2 interactions in Phragmoplastophyta might be associated with their preadaptation for water resilience in the terrestrial environment. Further experiments using the *ppein2ab* mutants will help us understand functions of EIN2 orthologs in various alga groups and determine domains critical for the submergence and osmostress responses.

## Materials and methods

### Plant materials and the chemicals

*Physcomitrium patens* protonemata were cultured on cellophane-overlaid BCDAT agar media under continuous light as described previously (Nishiyama et al. 2000). Chemicals were purchased from Fujifilm-Wako (Osaka Japan) except for the ABA, which was from Sigma (A4906, St. Louis, MO, USA).

### Molecular phylogenetic analyses

Sequences of EIN2-like homologs obtained from public databases were aligned with MAFFT v7.520 (Katoh and Standley 2013) with default parameters. Ambiguously aligned positions were filtered using trimAl v1.2 (Capella-Gutierrez et al. 2009) with the option “auto- mated1.” The aligned dataset (730 sites) was subjected to maximum likelihood (ML) analysis using RAxML-NG 1.2.2 (Kozlov et al. 2019). ModelTest-NG 0.1.7 (Darriba et al. 2020) was used to identify the sequence evolution model that fit the dataset using the corrected Akaike information criterion. The substitution model applied was LG + I + G4 + F. The bootstrap percentages (Felsenstein 1985) in the ML analysis were calculated based on 1,000 replicates. The phylogenetic tree was visualized in FigTree v1.4.4 (http://tree.bio.ed.ac.uk/software/figtree/).

### Genome editing of *P. patens*

Transformation of *P. patens* was carried out by the polyethylene glycol (PEG)-mediated method using protoplasts of protonemata (Nishiyama et al. 2000). The CRISPR-Cas9-mediated genome editing was carried out according to the protocol described by Lopez-Obando et al. (2016).

### Tests for freezing tolerance

Protonemata tissue placed in a glass tube with 500 µL water was ice-inoculated and kept at −1 °C for 1 h using a cooling bath. The program was set to decrease the temperature by 1 °C in every 40 min. When reached to the target temperature, the tube was taken out and thawed at 4 °C overnight and the electrolyte leakage was measured (Saruhashi et al. 2015; Islam et al. 2021).

### Tests for osmostress tolerance

Six-day-old protonemata were pretreated with 10 µM ABA or 0.5 m mannitol for 1 day and exposed to different concentrations of mannitol and NaCl as the stress treatments for 15 min. The protonemata were then grown on BCDAT media for 2 weeks to determine the survival.

### Reporter assays

Five-day-cultured protonema cells were bombarded with plasmids using the PDS-1000He particle delivery system (Bio-Rad, Hercules, CA, USA). For analysis of ABA-induced gene expression, the ABA-inducible *Em-*promoter fused to the *beta-glucuronidase* (*proEm-GUS*) was used as the reporter and the *Ubiquitin* promoter fused to *luciferase* (*proUbi-LUC*) was used as the reference, along with the *Actin* promoter-driven cDNA constructs fused to GFP as effectors. The bombarded cells were cultured with or without ABA for 1 day, and GUS and LUC activity was measured as described previously (Marella et al. 2006).

### RNA extraction for RT-qPCR analysis

The total RNA preparation and SYBR Green-based RT-qPCR were carried out according to the protocols described previously (Jahan et al. 2019). Nucleotide sequences of primers are listed in Supplementary Table S1.

### Protein extraction, electrophoresis, and immunoblot analysis

Protein extraction, SDS-polyacrylamide gel electrophoresis and electroblotting were performed following the protocols described by Islam et al. (2021). For analysis of boiling-soluble proteins, total soluble proteins prepared from the moss protonemata were boiled for 1 min and centrifuged at 20,000 × *g* for 5 min. The supernatant was used for gel electrophoresis, followed by Coomassie Brilliant Blue staining or immunoblot analysis using the anti-17B9 antibody (Islam et al. 2021).

### In-gel kinase assay for detection of SnRK2 activity

In-gel kinase assay was performed to detect the SnRK2 activity using the gamma-^32^P-ATP and Histone IIIS (H5055, Sigma, MO, USA) as described by Islam et al. (2021). In brief, total soluble proteins were electrophoresed in SDS-polyacrylamide gel containing Histone IIIS, and the gel was sequentially rinsed in 100 mm Tris-Cl (pH8.0) buffers containing 20% isopropanol for 1 h, 6 m guanidine hydrochloride for 1 h, and 0.04% Tween-40 and 5 mm beta-mercaptoethanol for 16 h. The gel was then reacted for 1 h with 50 µM cold ATP and 1.85 mBq of gamma-^32^P-ATP in a buffer containing 40 mm Hepes (pH7.5), 10 mm MgCl_2_, 0.1 mm ethylene glycol bis(beta-aminoethylether)-N,N,N,N-tetraacetic acid, and 1 mm dithiothreitol at 25 °C. After thorough washing in 5% (w/v) trichloroacetic acid and 1% (w/v) sodium pyrophosphate, the gel was dried and used for autoradiography to detect radioactive signals.

### Yeast two-hybrid assays

Yeast two-hybrid assays were carried out using the GAL4-AD (pGADT7) and GAL4-BD (pGBKT7) vectors (Clontech). Haploid yeast strains PJ69-4A-α and PJ69-4A-a carrying *P. patens* sequences cloned in these vectors were used for mating. To verify the activation of the *HIS3* and *ADE2* reporter genes in the transformed yeast cells, colonies were grown on synthetic defined (SD) media lacking leucine, tryptophane, histidine, and adenine (SD/-LWHA) (Toriyama et al. 2022).

### Accession numbers

Sequence data from this article can be found in the Phytozome/GenBank/EMBL data libraries under accession numbers_Pp3c16_16830V3.1 (PpEIN2A), PpEIN2B (Pp3c27_2050V3.1) (PpEIN2B), AT5G03280 (AtEIN2) and CHBRA353g00160 (CbEIN2).

## Supplementary Material

kiaf293_Supplementary_Data

## Data Availability

The data underlying this article will be shared on reasonable request to the corresponding author.

## Supplementary data

The following materials are available in the online version of this article.

**Supplementary Figure S1.** Generation of the *ppein2* genome-editing lines of *Physcomitrium patens*.

**Supplementary Figure S2.** Abscisic acid (ABA) responses in the independent *ppein2ab* lines.

**Supplementary Figure S3.** Abscisic acid (ABA) responses in the *ppein2a and ppein2b* single mutants.

**Supplementary Figure S4.** C-terminal and N-terminal deletion analysis of PpEIN2A.

**Supplementary Figure S5.** Amino acid sequence alignment of EIN2 orthologs.

**Supplementary Figure S6.** Freezing tolerance of protonemata with or without cold acclimation.

**Supplementary Figure S7.** Yeast two-hybrid assays of PpEIN2B.

**Supplementary Figure S8.** Submergence responses in the *ppein2a* and *ppein2b* lines.

**Supplementary Figure S9.** Expression of PpEIN2A-GFP restores ABA and submergence responses in *ppein2ab*.

**Supplementary Figure S10.** Generation of the *ppein3ab* and *ppein2/ppein3* genome-editing lines.

**Supplementary Figure S11.** Freezing tolerance is unchanged in *ppein3ab* mutants.

**Supplementary Figure S12.** Expression of submergence-responsive genes in the *ppein3ab* lines.

**Supplementary Figure S13.** Reporter assays with the full-length and C-terminally truncated *PpEIN2B* constructs.

**Supplementary Figure S14.** Localization of GFP fused to PpEIN2A and PpEIN2B after abscisic acid (ABA) treatment.

**Supplementary Figure S15.** Effect of *PpEIN2_CEND* expression on abscisic acid (ABA)-induced gene expression in *ark*/*ctr1 l* and *pphkQKO*.

**Supplementary Figure S16.** Measurement of PpHK5-LUC activity in wild type (WT) and *ppein2ab*.

**Supplementary Table S1.** Primers used for the RT-qPCR analysis.

## Dive Curated Terms

The following phenotypic, genotypic, and functional terms are of significance to the work described in this paper:

ABI3 Gramene: AT3G24650
ABI3 Araport: AT3G24650

ABI5 Gramene: AT2G36270
ABI5 Araport: AT2G36270

EBF1 Gramene: AT2G25490
EBF1 Araport: AT2G25490

EBF2 Gramene: AT5G25350
EBF2 Araport: AT5G25350

EIN4 Gramene: AT3G04580
EIN4 Araport: AT3G04580

ERS2 Gramene: AT1G04310
ERS2 Araport: AT1G04310

ETR2 Gramene: AT3G23150
ETR2 Araport: AT3G23150

role CHEBI: CHEBI:50906

ERS1 Gramene: LOC_Os10g22380
ERS1 Araport: LOC_Os10g22380

EDR1 Gramene: AT1G08720
EDR1 Araport: AT1G08720

EIN2 Gramene: AT5G03280
EIN2 Araport: AT5G03280

CTR1 Gramene: AT5G03730
CTR1 Araport: AT5G03730
