# Supplementary material for: ETHYLENE INSENSITIVE2-like protein mediates submergence and drought responses in Physcomitrium patens
Source: Plant Physiol. 2025 Jul 2;198(3):kiaf293. doi: 10.1093/plphys/kiaf293 (PMC12290401; doi:10.1093/plphys/kiaf293)
Supplement: kiaf293_Supplementary_Data [file kiaf293_supplementary_data.pdf]

(a)

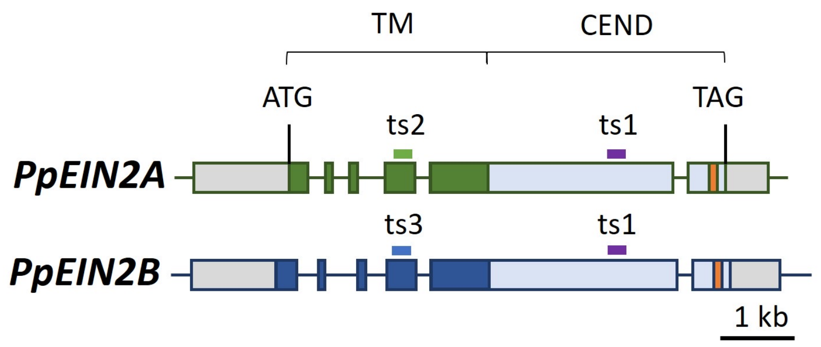

(b)

	PpEIN2A	***PpEIN2B***
**WT**	CCTGAGCGCCAGTATTCCAGCATGC (ts1)	CCTGAGCGCCAGTATTCCAGCATGC (ts1)
ppein2ab_1	CC----------------------------AGCATGC	CC--------------AGTATTCCAGCATGC
**WT**	GAGACTCCCTCTACATCGCTGTGGGACTGA (ts2)	CGCATTCCTTGGTGGCGTGTGGCCCA (ts3)
ppein2ab_38	GAGACTCCCTCTACATCGC(AT inserted)TGTGGGACTGA	CGCATTCCTTGGTGGC-------------CCA
ppein2ab_39	GAGACT--------------------------------------GA	CGCATTCCTTGGTGGC-------------CCA
ppein2a	GAGACTCCTTCTACA-------TGTGGGACTGA	CGCATTCCTTGGTGGCGTGTGGCCCA
ppein2b	GAGACTCCCTCTACATCGCTGTGGGACTGA	CGCATTCCTTGGTG----------TGGCCCA

Supplementary Figure S1. Generation of the *ppein2* genome-editing lines of *Physcomitrium patens.* (a) Gene structures of *PpEIN2A* and *PpEIN2B* with the marked positions of 19-nucleotide target sequences ts1, ts2 and ts3 for genome editing. The nucleotide sequence of ts1 is common in *PpEIN2A* and *PpEIN2B*. (b) Deletions and additions of nucleotides found in the *ppein2ab* double knockout lines *(ppein2ab_1, ppein2ab_38 and ppein2ab_39*), and single disruptants *ppein2a* and *ppein2b. ppein2ab_1* has 16-bp and 8-bp deletions in *PpEIN2A* and *PpEIN2B*, respectively; *ppein2ab_38* has a 2-bp addition in *PpEIN2A* and a 7-bp deletion in *PpEIN2B*; *ppein2ab_39* has 22-bp and 7-bp deletions in *PpEIN2A* and *PpEIN2B*, respectively. The single disruptants *ppein2a* and *ppein2b* have a 4-bp deletion in *PpEIN2A* and a 5-bp deletion in *PpEIN2B*, respectively.

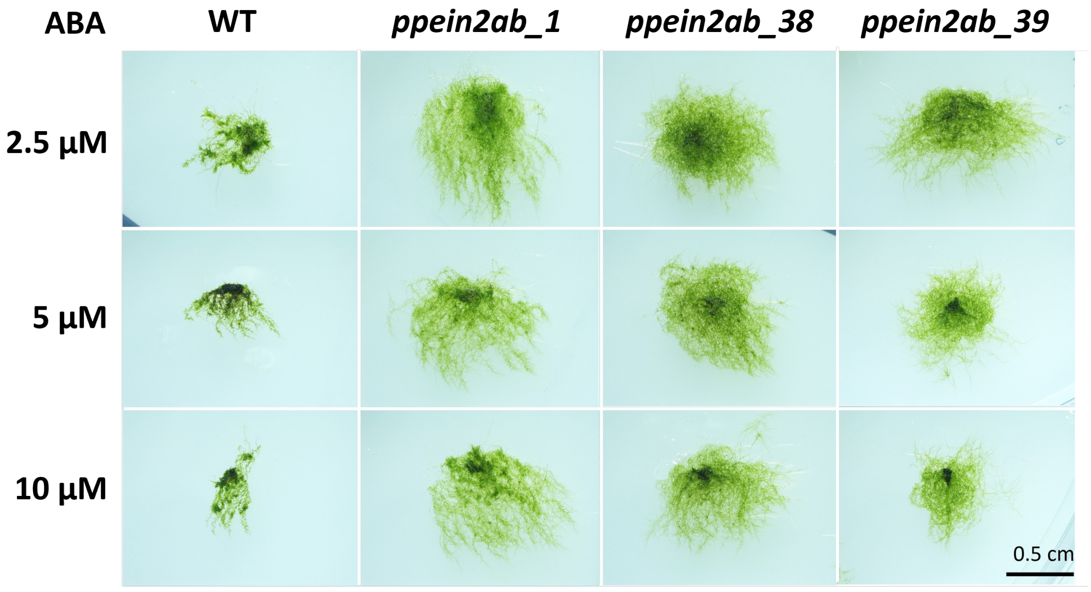

Supplementary Figure S2. Abscisic acid (ABA) responses in the independent *ppein2ab* lines. Growth of protonemata of wild type (WT) and the *ppein2* genome editing lines. The protonemata were cultured with the indicated concentrations of ABA for two weeks. The scale bar is 0.5 cm applicable for all colonies.

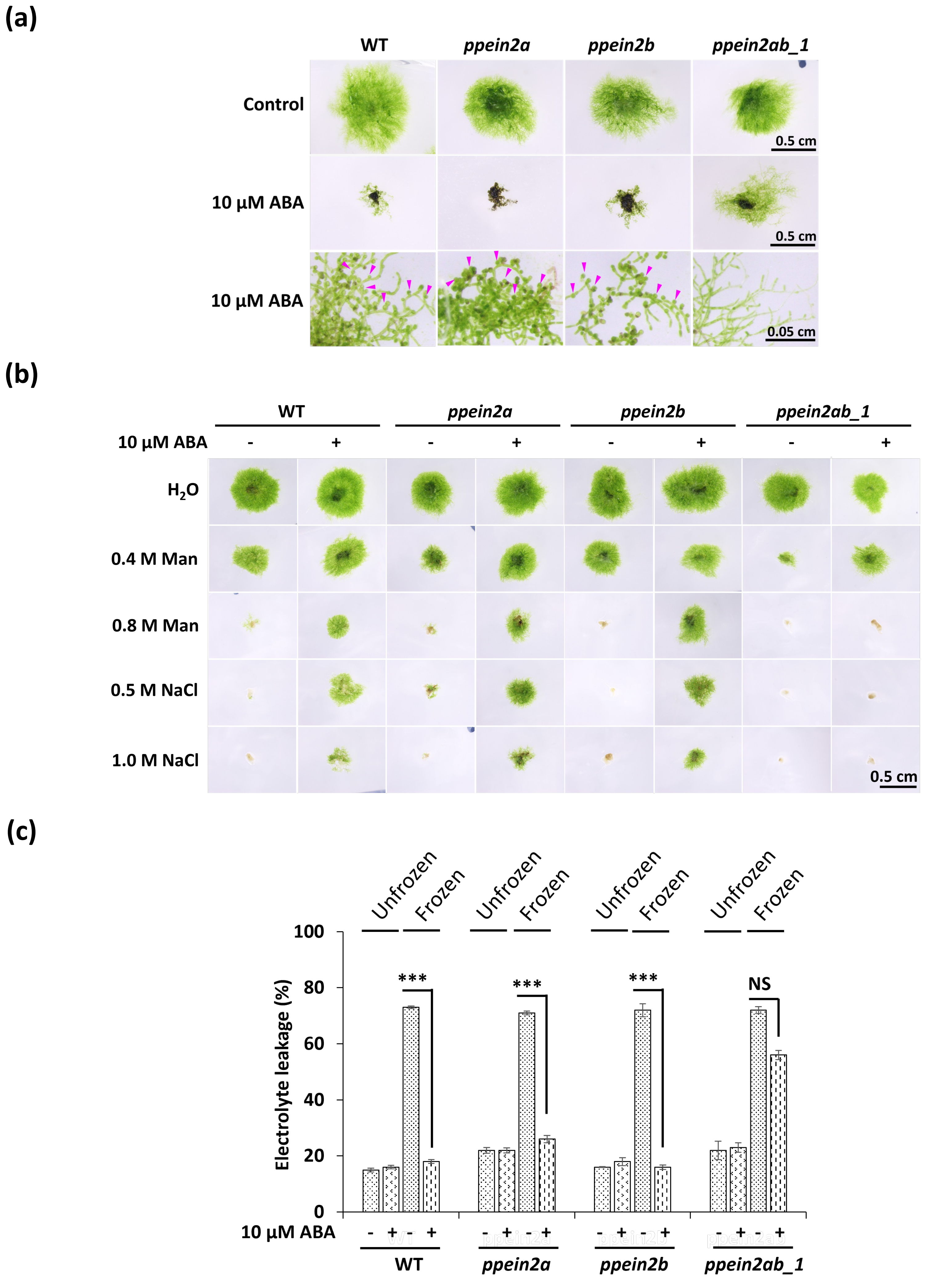

Supplementary Figure S3. Abscisic acid (ABA) responses in the *ppein2a* and *ppein2b* single mutants.(a) Effect of ABA on protonemal growth of wild type (WT), *ppein2a*, *ppein2b* and *ppein2ab_1*. Plants were grown in medium with or without the 10 µM ABA for two weeks. The magenta arrowheads indicate the brood cells. (b) Tests for osmostress tolerance. Cultured protonemata were pretreated with or without 10 µM ABA for one day and exposed to the indicated concentrations of mannitol (Man) and NaCl for 15 min for osmostress treatment. Protonemata were then cultured in normal growth media for 10 days to determine survival. For (a) and (b), the scale bars are applicable for other images. (c) Tests for freezing tolerance. Protonemata were pretreated with 10 µM ABA and either kept unfrozen or frozen to -4°C. Electrolyte leakage was measured after thawing to determine freezing injury. Error bars indicate SE (n=3). ***p<0.001 by Student's t-test; NS, not significant.

(a)

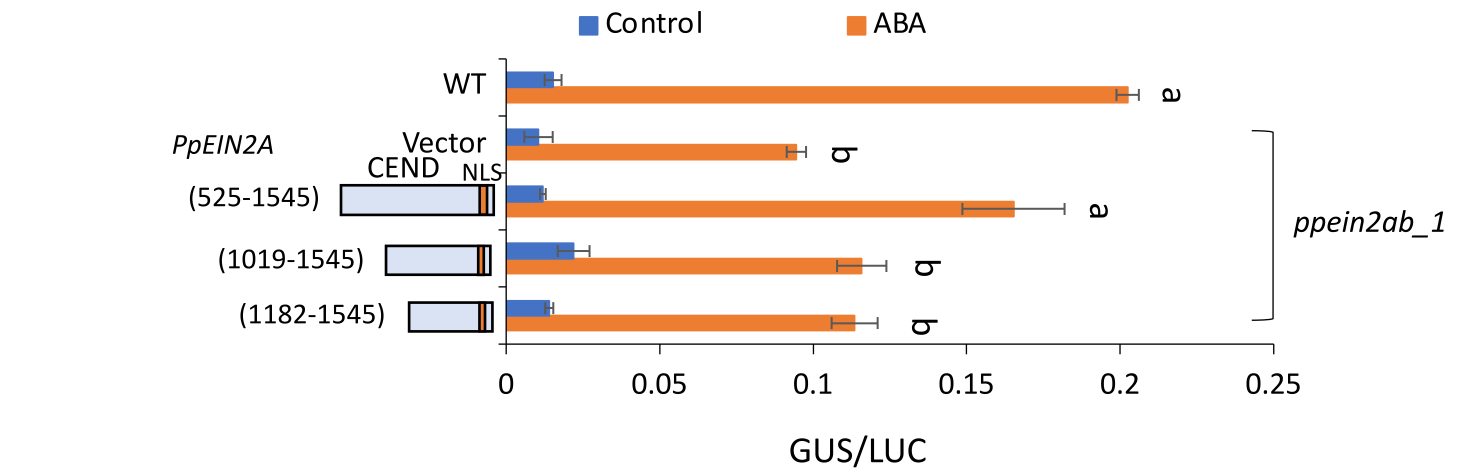

(b)

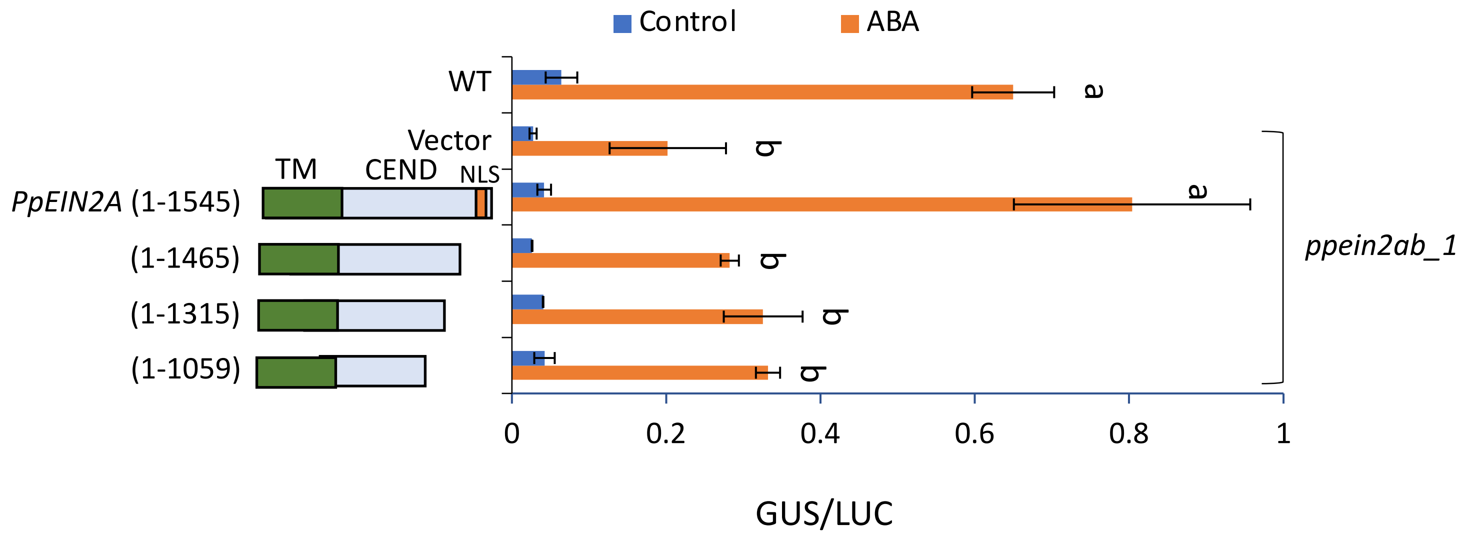

Supplementary Figure S4. C-terminal and N-terminal deletion analysis of PpEIN2A. (a) C-terminal and (b) N-terminal deletion constructs of *PpEIN2A* introduced into *ppein2ab_1* to evaluate the abscisic acid (ABA) response recovery. The procedures are same as stated in the main figures. The protonemata were cultured for one day with or without 10 µM ABA and activity of GUS and LUC was determined. The results were analyzed in one-way ANOVA among the ABA treated samples. The error bars represent SE (n=3). The different letters denote statistical difference ($p<0.05$).

```
PpEIN2A     MDSSPSVPKWMDPSSSMAKHVPALAPIILVAIGYMDPGNWACAIEGGSRFGFELLWVVIL
CbEIN2      -------MRRSVGQSAFAKVVPFVGPAFLVSVGYMDPGNWATAIEGGSRFGYELAWVVVL
AtEIN2      --MEAEIVNVRPQLGFIQRMVPALLPVLLVSVGYIDPGKWVANIEGGARFGYDLVAITLL
                      .     . : : ** : * :**::**:***:*.  ****:***::*  :.:*

PpEIN2A     SNCMAAFFQTLATRLGLVSGKHLAEICREDYPRPVCLLLWMLSEASIIALDLTMVIGAAT
CbEIN2      SNIIAILLQTLSARLGLVTGKHLAQVCRDEYPHGVCILLWILCEVSIVALDLTMVLGTAI
AtEIN2      FNFAAILCQYVAARISVVTGKHLAQICNEEYDKWTCMFLGIQAEFSAILLDLTMVVGVAH
             *  * : * :::*:.:*:*****:*.::* : .*::* : .* * : ******:*.*

PpEIN2A     GLNLLFGFPLLPSVLLLSVDGIILMVALPMVGVHRAEMVAGTIVGVVLVCFGLGALLSGP
CbEIN2      GLNLLFGIPMLPGVLLMGLDALLILVVLPHLGVRKAEIATVAVVGIVLLCFVVDIFLSKP
AtEIN2      ALNLLFGVELSTGVFLAAMDAFLFPVFASFLENGMANTVSIYSAGLVLLLYVSGVLLSQS
            .******. : ..*:* .:*.::: *   .  :    *: .:    .*:**: :  . :** .

PpEIN2A     TAPAIISGLWPKVRGDSLYIAVGLIGANVMPHTFYLHSALLQGQHKLSSKAKEIVVQENM
CbEIN2      PMRAVVSGLWPRLSRDSLYTAVSLLGANVMPHNFYLHSALVKGQVKAGEKSVGALCYYNF
AtEIN2      EIPLSMNGVLTRLNGESAFALMGLLGASIVPHNFYIHSYFAGESTSSSDVDKSSLCQDHL
                 :.*: .::  :* :  :.*:**.::**.**:** :    . . ..    :   ::

PpEIN2A     RDTIGAFGVAMLANVAVLMVAASVFHNVGLVILTLQDAHALMEQILSNSVAPAVFGLALL
CbEIN2      LDITCALGAALLVNLAVLIVAAATFHSEGLVVLTLQDAHALMEQILSNSMAPLAFGLALL
AtEIN2      FAIFGVFSGLSLVNYVLMNAAANVFHSTGLVVLTFHDALSLMEQVFMSPLIPVVFMLLLF
                 .:.   *.* .:: .** .**. ***:**::** :****:: ..: * .* : *:

PpEIN2A     CAGQLSSLSGTLTGKVTMEGFLDMSMRPWLHRSLIRGAAVIPAAFCAWHYGSEGLYRLLL
CbEIN2      SAGQLSTLTGTIAGQVVLEGFLGYEMRAWLRRVLTRSAAIIPAALYIWRYGDEGTYRMLV
AtEIN2      FSSQITALAWAFGGEVVLHDFLKIEIPAWLHRATIRILAVAPALYCVWTSGADGIYQLLI
                :.*:::*: :: *:*.:..** .: .**:*   *  *: **    *  * :* *::*:

PpEIN2A     FAQIVIAVELPFSAVPLVKASASESRMGSYKIPSLVESIAWVSVALVVVANIWMVFDVLL
CbEIN2      LMQVVLALQLPFTIVPLIKVTSSKGYMGVHKNPKLVEVLAWASSGLVFFINVWLVVDMFI
AtEIN2      FTQVLVAMMLPCSVIPLFRIASSRQIMGVHKIPQVGEFLALTTFLGFLGLNVVFVVEMVF
            : *:::*: ** : :**.: ::*.  **  :* *.: * *:  * :* .:   ..  *: :*.::.:

PpEIN2A     DEIDEFSGFAAHLESLLGTDSFGHHGEDTLNTSVFALVLIVIGLSVG---FLIWLVVTPL
CbEIN2      GKGDHTPLSGKSQVAHAGMAFKPMAASSKKDASLEHLQGVGVVGLVAERDGFVLAVDGPS
AtEIN2      GSSDWAGG----------------------------------------------------
            .. *

                                                            525
PpEIN2A     RVDRIAMERKWVQEYELFESQTLSDDRLGVLEYSSDPTTSEVYDMIPMTEDIMLVNNLVG
CbEIN2      LFGFPRQDMVQAIGEMMLLITALSGAAVGVVLLGWMIMVPLKGNERVHNDGEQEFGKKKG
AtEIN2      ------LRWNTVMGTSIQYTTLLVSSCASLCLILWLAATPLKSASNRAEAQIWNMDAQNA
                         .    :     * .   .:         ..               ..   .

PpEIN2A     LEYPSRQESALSSIDTDPALELGRGELVKWTSTVSTSSSDIDDIVSNQPELVYLSEPAQL
CbEIN2      REDWWLESDQRTKESDKEATELRPLELIEVG------LREPQDQDVWEKSPRYVVDVDVD
AtEIN2      LSYPSVQEEEIERTETR------------------------RNEDESIVRLESRVKDQLD
             .    :..     .                          :                .

PpEIN2A     NGLVRTSELNEAAEPFIPSGKSLSSEPVLTSPHLPHESDKETPLKVVDPPVDATVFNQLR
CbEIN2      ADDKDRDGVGETRAELVGAGREHATEKVEEEGDKKLLIELSGVGRPHPPGRRDWKGRDSR
AtEIN2      TTSVTSSVYDLPENILMTDQEIRSSPPEERELDVKYSTSQVSSLKEDSDVKEQSVLQSTV
               . . .   ::    .   .   .   .           :                ..
                                                                 711
PpEIN2A     VPAYSKVVSHDLNVIAKPVDDSMVVASLPSVVDEKKQDVVQEAPFGHDRGHILTEPEIAG
CbEIN2      RAHQRDREKEKQGSEGIGGGNAGLRDREKDKGDDGDGGGGRGGGQLGWLYDLIHESMFMY
AtEIN2      VNEVSD------------------------------------------------------
                    .

PpEIN2A     TIETFLKDASLSGDGLTKKSTAPPPQRLVAVRRHEPLAADEAESIALKRAEAEADADLID
CbEIN2      CINSGHLKGWGPADQEPDVSSCPRPYAVKESVAWRPPNASKTP-CTLGRSGRRQFASILD
AtEIN2      ---------------------------KDLIVETKMAKIEPMSPVEKIVSMENNSKFIE
                                                    :.   .       .  :.:::
                                                               831
PpEIN2A     KDDDEVDDFDHEDVVVGPLSHDNLRRSYSNLAYEASGSARSLGGRTDASEGGGSGSGSLS
CbEIN2      DFWAHIFDLHGQPAGPLGVWLLGFGGSGGGGGGGGGGGGAVGGGDGGDDGGGDDQRAIP
AtEIN2      KDVEGVSWETEEATKAAPTSNFTVGSDGPPSFRSLSGE-------------GGSGTGSLS
            .    :    : .         .  .      .*           **..   ::.
                                              870
PpEIN2A     RLSGLGRAARRQFAAYLDEFWGKLFDLHGQPIASKGSTRLSALGSSIGPLSLPFDNPGIT
CbEIN2      DQN---RSDARPIGTDKRGHVREYKEQTMLPVESASNGLLSIEGDRSLGKPLPTSSGGAS
AtEIN2      RLQGLGRAARRHLSAILDEFWGHLYDFHGQLVAEARAKKLDQLFGTDQKSASSMKADSFG
              .   *:  * :.:     .   .  :      : .      *.    .       . . .  .
                                                                  947
PpEIN2A     PRSGDPGAYLG----------RLNSPPDGYLGGESNGYGSKRGPSKSTSSLHQQVEQHFM
CbEIN2      TYVPWHGYSVGGQPISAPMVSKDQYATENMYQNQTKGNNWEVGQHRTSDGCGSNQGKDSA
AtEIN2      -----------------------KDISSGYCMSPTAKGMDSQMTSSLYDSLKQQRTPGSI
                                   :  ...   . :      .        ..  .:

PpEIN2A     DAYARAQS-----HATATTSSGFLGFMHDYSGRSGNSGSLQLPERQYSSMRLPTFSDEFD
CbEIN2      DGIVSKSAPKVLVFDDCAPQVPAFQPFSLFSNPLGSHGTQIVPSPRDSSIAAGFTGPFVQ
AtEIN2      DSLYGLQR-----GSSPSPLVNRMQMLGAYGNTTNNNNAYELSERRYSSLRAPSSSEGWE
            *.     .            :.    :  :  :..  .. .:  :.. : **:      .   :

PpEIN2A     REPATIHGYYAPSFLGGRSATSSPALKSTRPMSSLDMHGAASARALSGQLHVQQPLGVGV
CbEIN2      WDRAEVADGRNPTQTAATGSVIREGSQRRWNLGDPLLVVDSVRKTQMQPQHIQPYGGGLW
AtEIN2      HQQPATVHG-----YQMKSYVDNLAKERLEALQSRGEIPTSRSMALGTLSYTQQLALALK
             : .               .  .    . :     : .       :    :       : *

PpEIN2A     QGEFEGSLYG-----GGFQGSLEDAIQSLRSMHALPSRN--AMNCKVNADRELNGPRSWD
CbEIN2      GLERVPSTYHNWRQEGGFLGGDSRARESSVESMSRQESNPESLSYWLRSPQEVLFPSPST
AtEIN2      QKSQNGLTPGPAPGFENFAGSRSISRQSERSYYGVPSSGNTDTVGAAVANEKKYSSMPDI
              .             .* *. . : :*  .  .  .  .            : .:    .  .

PpEIN2A     SRMATVDPLVHRATGEIYQPSSGHSPIGRIGSMSSVRAPLSFDELSPSVTHRDGFSIQTA
CbEIN2      PSQNIGGAGVQPIAAAEPSTLWHHSPSQLDGSLAALKTAAGIPTAAG-IPGYEEDMISSA
AtEIN2      SGLSMSARNMHLPNNKSGYWDPSSGGGGYGASYGRLSNESSLYSNLG------SRVGVPS
            .         ::               .      .* . :     .:                .:

PpEIN2A     QSENSLWSRQPFEQSFSGMTDPSANGGSSRSRGGSGGLGTNRNGTVGGRPGKTSGGAESL
CbEIN2      PEDFAHRSTQPVADAAAQPASRTNKGGESRVSVPSFTATTDPLDAILDELDLCLEKLRQL
AtEIN2      TYDDISQSRGGYRDAYSLPQSATTGTGSLWSRQPFEQFGVAERNGAVGEELRNRSNPINI
              :     *      :: :    . :    *.              .    .    ..            .:

PpEIN2A     SNASTAFSSGLDADLDMMETLRACICKLLCLEGSEWLFRFDNGSDEDLVAAVAAVEKMHM
CbEIN2      DKMEWLFRLRFSPDEVVMGWLGFSWVGNGPSDDGNQLDSTVIAEPIHSAGAVLPGPDTI
AtEIN2      DNNASSN---VDAEAKLLQSFRHCILKLKLEGSEWLFGQSDGVDEELIDRVAAREKFIY
            .:          ...:  ::  :  .          :..: *      . :        ..

PpEIN2A     DADSPDRVSRNERWQAHG-----------------RPVSSFLKGSNDARRVCYCGEACVW
CbEIN2      IVRQPEAALTPHREDRIGGCLDAQGSLCQAPTPQEGRRFKAARSRARVRKRGCGEKHCVW
AtEIN2      EAEAREINQVGHMG------------------------------EPLISSVPNCGDGCVW
             .   :      .                                                 . ***

PpEIN2A     GKGLLISFGVWCVHRVLELSLMESRPELWGKYTYVLNRLQGVLDPAFSKPRLVAPMCNCV
CbEIN2      NRDLIVSFGVWCVLRMLELCQLESRPELWGKYTYVLNRLQGILESAFLVPRRGPPTCLCI
AtEIN2      RADLIVSFGVWCIHRVLDLSLMESRPELWGKYTYVLNRLQGVIDPAFSKLRTPMTPCFCL
              .*::******: *:*:*. :******************::.**   *    . * *:

PpEIN2A     IEVSEILSRR-----------------------------APRGGLIK------------QG
CbEIN2      RNPLDSPLLPGAWDIRKPYPPPSPPPRDSHPASSRPKDPPQHGQTPGNCSEQTPLGHDRS
AtEIN2      QIPASHQRAS--------------------------------------------------
                .
                                                                  1465
PpEIN2A     SNGFRIDSTNGDHVSLPQSSYPFPWSWGRNSSSSKGRGAS----ATVFLEMIREVEQAVG
CbEIN2      SQSLEEERRDTDMITCPLAVLAIIVAQTRSLPDVRLFAPDNASLISSVLDLIKSIEIAVG
AtEIN2      -----------------------PTSANGMLPPAAKPAKGKCTTAVTLLDLIKDVEMAIS
                                    :      .     . .       .*::*:.:* *:.

PpEIN2A     SRKGRTGTAAGDVAFPKGKENLASVLKRYKRRLSNKPPGAAGGGGGGGGGGGGRRSNNG
CbEIN2      SRKGRSGTAAGDVAFPKGKENLASVLKRYRRRIAGGSSTYSTTKAVAEVGLKGRRSLVMS
AtEIN2      CRKGRTGTAAGDVAFPKGKENLASVLKRYKRRLSNKPVGMNQDGPGSRKNVTAYGSLG--
            .****:***********************:**:. .              .  .  .

PpEIN2A     FAQALVFAACL-------------
CbEIN2      SAVFFFILVSSTFVFIPSQKFPIF
AtEIN2      ------------------------
```

Supplementary Figure S5. Amino acid sequence alignment of EIN2 orthologs. Sequences of PpEIN2A, Arabidopsis EIN2 (AtEIN2), and the EIN2 ortholog of *Chara braunii* (CbEIN2) are aligned by using the ClastalW alignment tool. Asterisks and dots indicate identical and similar amino acids among the polypeptides. The putative nuclear localization signal (NLS) sequences are marked in magenta. Deletion end points of PpEIN2A constructs shown in Fig. 3 are highlighted in bright green.

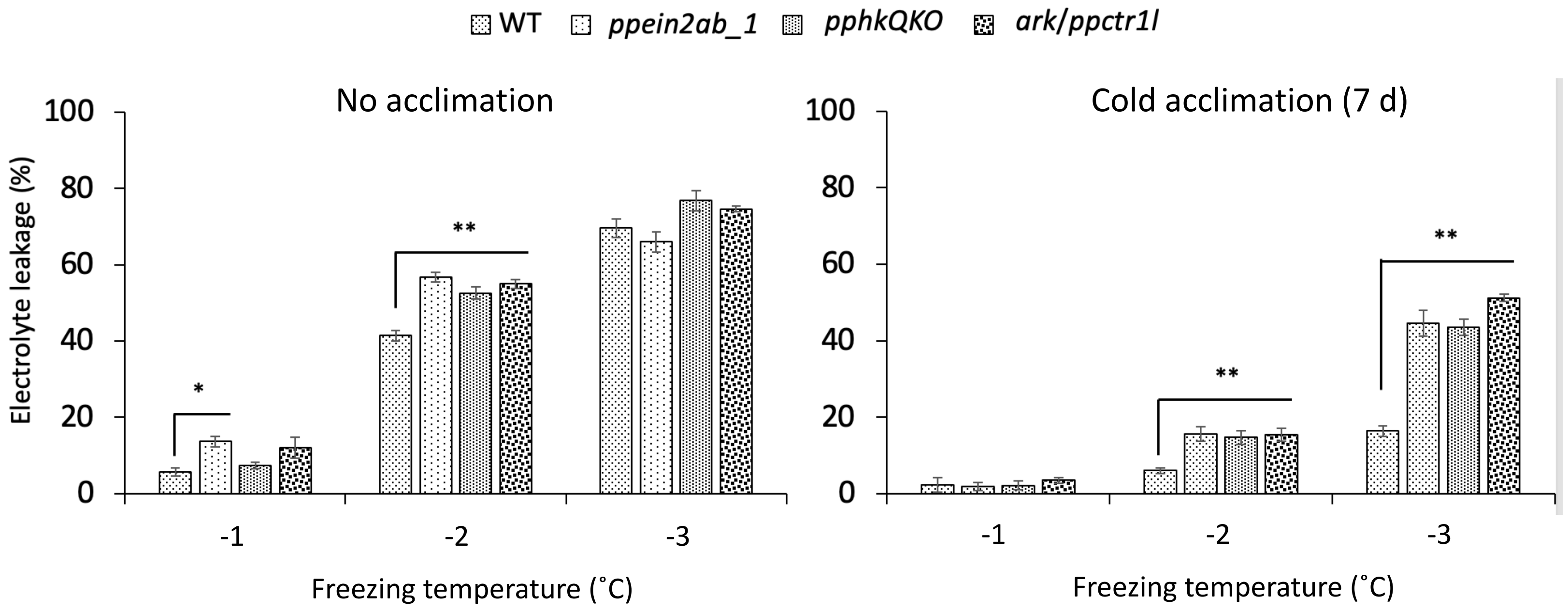

Supplementary Figure S6. Freezing tolerance of protonemata with or without cold acclimation. Protonemata of wild type (WT), *ppein2ab_1*, *pphkQKO* and *ark/ppctr1l* cold acclimated or without acclimation were subjected to freezing at the indicated temperatures. After thawing, the electrolyte leakage was measured to estimate freezing injury. The error bar represents standard error (n=3). Comparison is made against values of cold acclimated WT protonema using the Student's t-test (*p<0.05, **p<0.01).

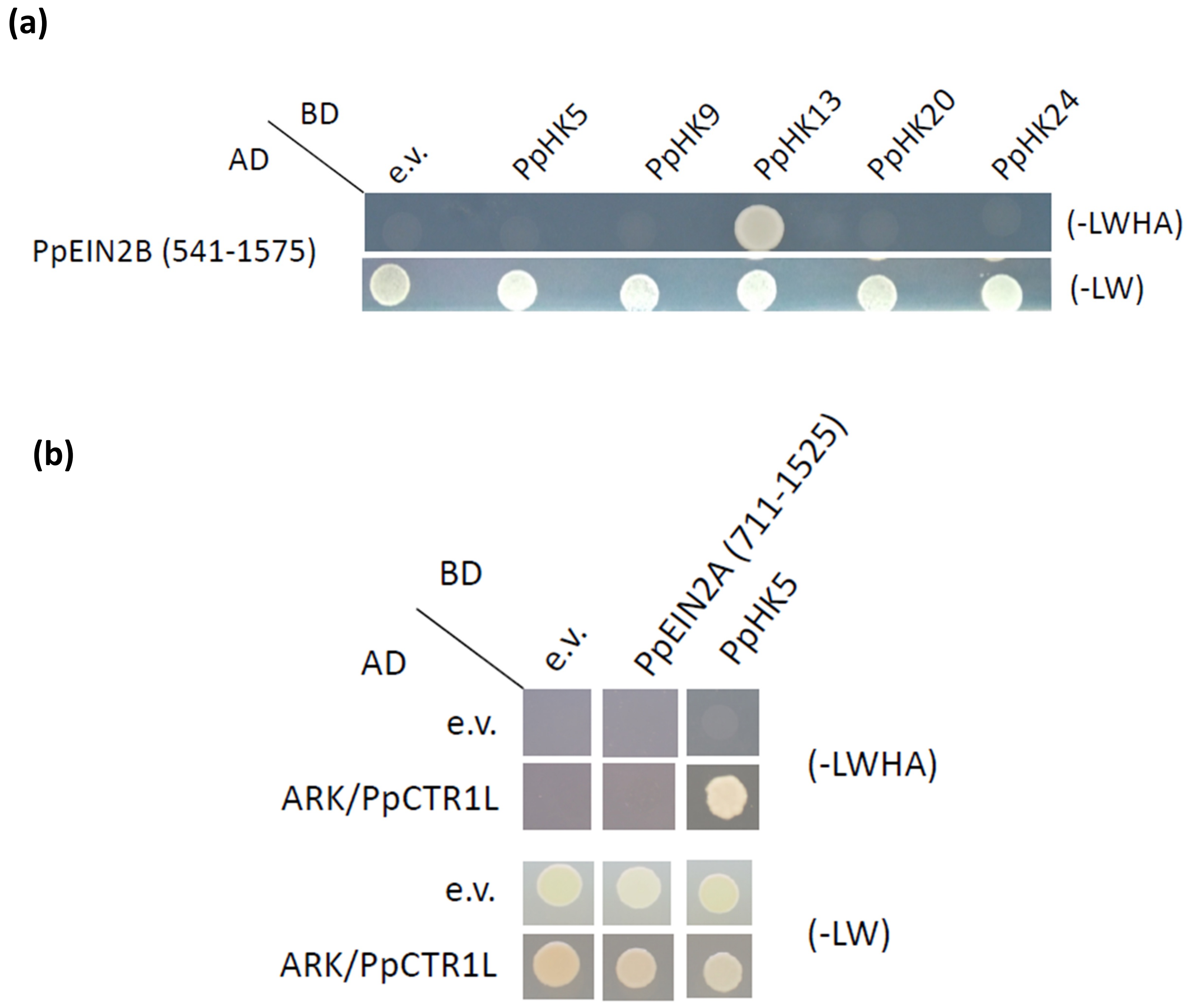

Supplementary Figure S7. Yeast two-hybrid assays of PpEIN2B. Haploid yeasts carrying the GAL4 activation domain (AD) fusion constructs and those carrying the GAL4 binding domain (BD) fusion constructs were mated and grown on the SD medium lacking Leu/Trp/His/Ade (-LWHA) or Leu/Trp (-LW). (a) Interaction of PpEIN2B with five ETR-HKs, PpHK5, PpHK9A, PpHK13, PpHK20 and PpHK24. (b) Interaction of ARK/PpCTR1L with PpEIN2A and PpHK5. Results are compared with those of the empty vector control (e.v.).

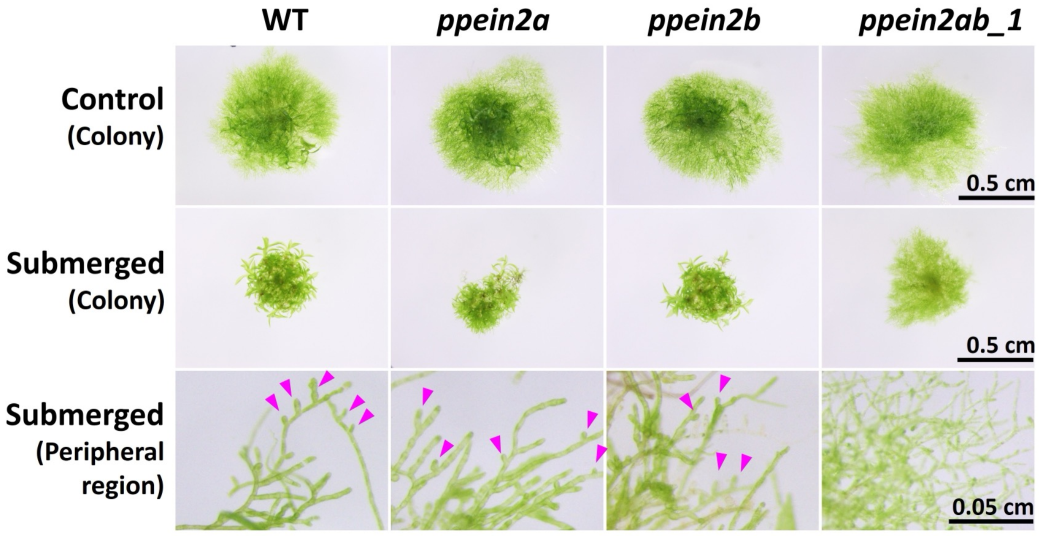

Supplementary Figure S8. Submergence response in the *ppein2a* and *ppein2b* lines. Submergence response in protonemata of wild type (WT) and the single disruptants *ppein2a* and *ppein2b*. Cultured protonemata were spotted on agar media and cultured for two days. Then, 30 mL of sterile water was gently added to each plate and further cultured for 3 weeks under continuous light. The magenta arrowheads indicate the formation of short branch under submergence. The scale bars on the rightmost images are applicable for other images.

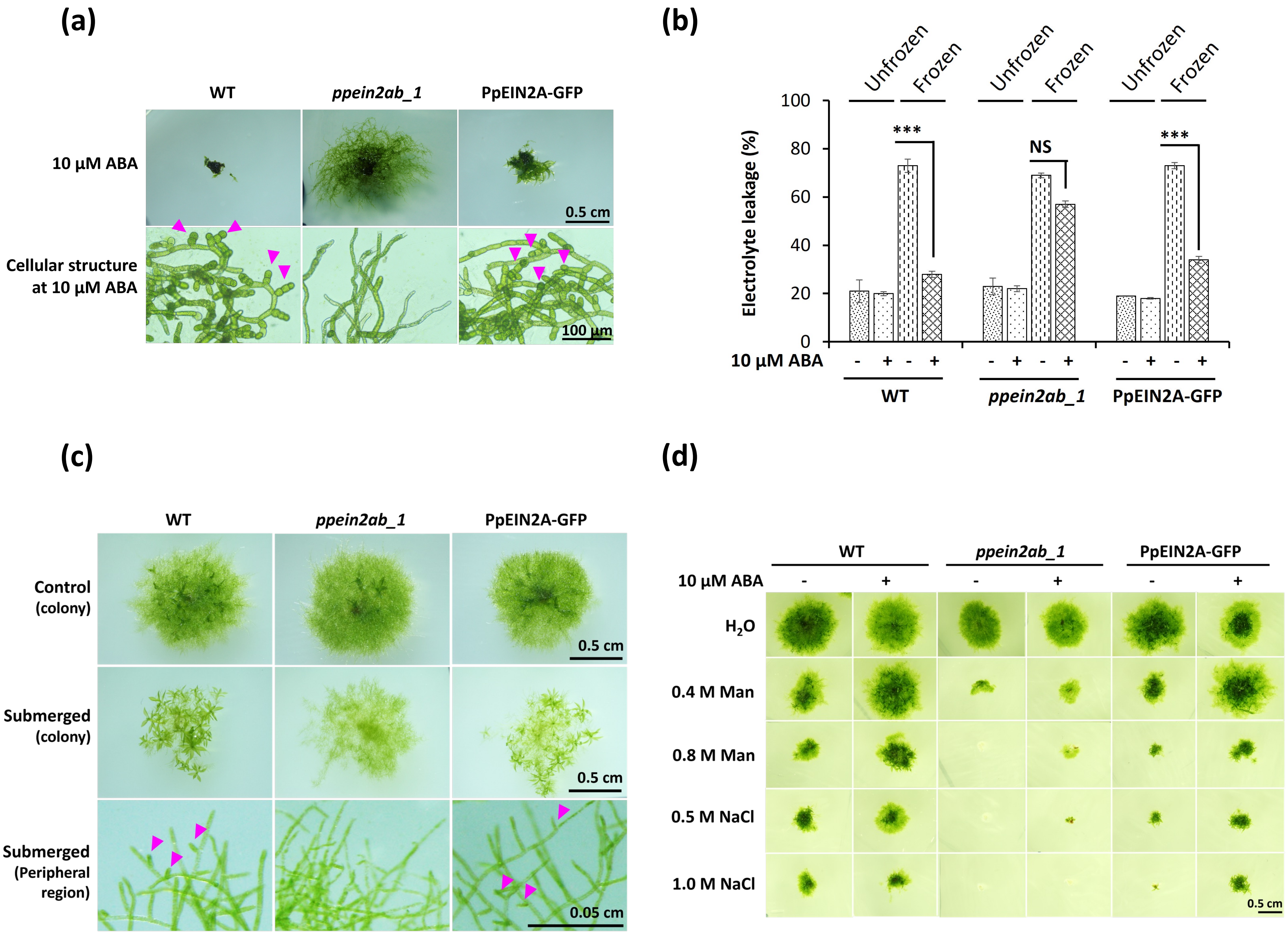

Supplementary Figure S9. Expression of PpEIN2A-GFP restores abscisic (ABA) and submergence responses in *ppein2ab*.(a) Growth analysis of WT, *ppein2ab_1* and *ppein2ab_1* expressing PpEIN2A-GFP with 10 µM ABA application. The magenta arrowheads indicate the formation of brood cells, which are not present in *ppein2ab_1* but found in WT and the *PpEIN2A-GFP* complementation line. (b) Test for freezing tolerance in the WT, *ppein2ab_1* and the *PpEIN2A-GFP* complementation lines. Protonemata were pretreated with 10 µM ABA and either kept unfrozen or frozen to -4°C. Electrolyte leakage was measured after thawing to determine freezing injury. Comparison is made against cold acclimated WT protonema separately using the Student's t-test (n=3, ***p<0.001). NS denotes non-significant. (c) Growth response analysis under submergence. Cultured protonemata were spotted on agar media and cultured for two days. Then, 30 mL of sterile water was gently added to each plate and further cultured for 3 weeks under continuous light. The magenta arrowheads indicate the formation of short branch under submergence. The scale bars on the rightmost images are applicable for other images. (d) Tests for osmostress tolerance with 10 µM ABA pretreatment. Cultured protonemata were pretreated with 10 µM ABA for one day. Both control and pretreated protonema were exposed to different concentrations of mannitol (Man) and NaCl for 15 min as osmostress, and then cultured in normal growth media for 10 days to determine survival. The scale bar is applicable for other images.

(a)

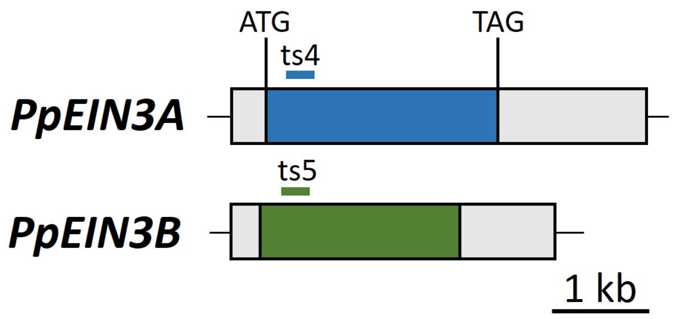

(b)

	PpEIN3A	***PpEIN3B***
**WT**	GGCGGTGGTGTCGGCGAGAGAGGGGTGGCGTCGGCGAAA (ts4)	GAGCCAGGAGCAGGCTAGGCGGAAGAAGATGAGT (ts5)
ppein3ab_2	GGCGGTGGTGTC--GGCGAAA	GAG--T
ppein3ab_5	GGCGGTGGTGTCGGCGA--A	GAG--T

(c)

	PpEIN2A	***PpEIN2B***
**WT**	CCTGAGCGCCAGTATTCCAGCATGC (ts1)	CCTGAGCGCCAGTATTCCAGCATGC (ts1)
ppein2/ppein3	CC--------------------------------AGCATGC	CC----------------AGTATTCCAGCATGC

	PpEIN3A	***PpEIN3B***
**WT**	GGCGGTGGTGTCGGCGAGAGAGGGGTGGCGTCGGCGAA (ts4)	GAGCCAGGAGCAGGCTAGGCG (ts5)
ppein2/ppein3	GGCGGTGGTGTCGGCGA---A	GAGCCAGGAGCAGGC---------G

Supplementary Figure S10. Generation of *ppein3ab* and *ppein2/ppein3* genome-editing lines. (a) Gene structures of *PpEIN3A* (*Pp3c7_9970V3.1*) and *PpEIN3B* (*Pp3c11_15260V3.1*) with the marked positions of 19-nucleotide target sequences ts4 and ts5 for genome editing. (b) Deletions and additions of nucleotides found in *ppein3ab_2* and *ppein3ab_5* are shown. (c) Deletions of nucleotides found in the *ppein2/ppein3* line are shown. The *ppein3ab_2* line has 20-bp and 30-bp deletions in *PpEIN3A* and *PpEIN3B*, respectively, and the *ppein3ab_5* line has 15-bp and 30-bp deletions in *PpEIN3A* and *PpEIN3B*, respectively. The *ppein2/ppein3* quadruple mutant has 16-bp and-8 bp deletions in *PpEIN2A* and *PpEIN2B*, respectively, and 20-bp and 5-bp deletions in *PpEIN3A* and *PpEIN3B*, respectively.

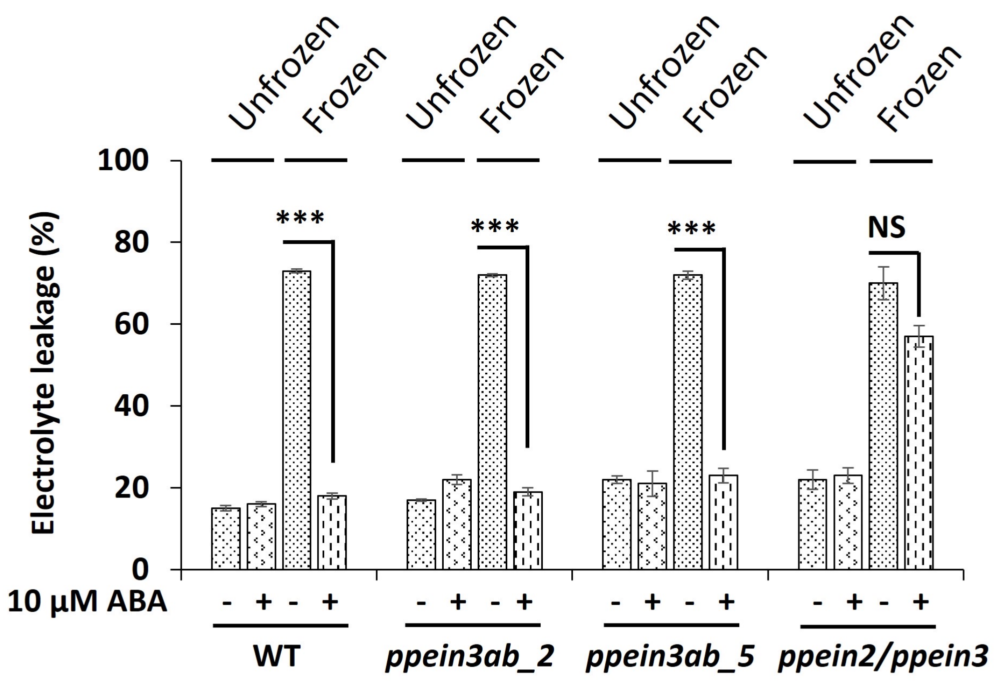

Supplementary Figure S11. Freezing tolerance is unchanged in *ppein3ab* mutants. Tests for freezing tolerance. Protonemata were pretreated with or without 10 µM abscisic acid (ABA) and either kept unfrozen or frozen to -4˚C. Electrolyte leakage was measured after thawing to determine freezing injury. Error bars indicate standard error (n=3). ****p*<0.001 by Student's t-test; NS, not significant (*p* >0.05).

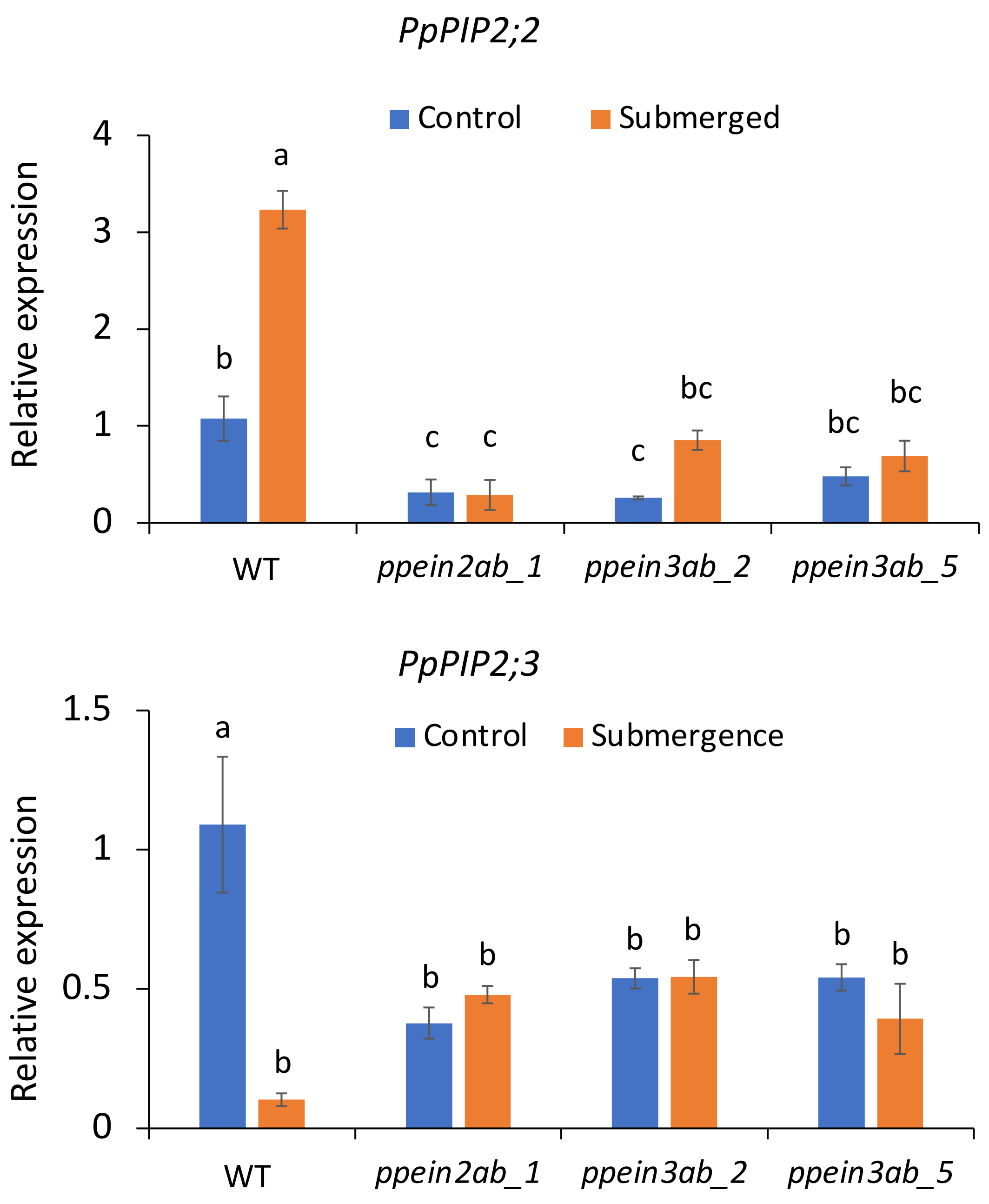

Supplementary Figure S12. Expression of submergence-responsive genes in the *ppein3ab* lines. Reverse transcription-quantitative PCR (RT-qPCR) analysis for submergence-responsive gene expression. The *Physcomitrium patens* protonemata grown for 7 days on agar media were completely submerged under water for three days. RNA extracted from the protonemata was reverse-transcribed and analyzed by the SYBR Green-based qPCR method with the *Tubulin* gene as the internal control. Standard error (SE) of means (n=4) is indicated. One-way analysis of variance (ANOVA) was performed to compare the means among different groups. Different letters represent the statistical difference (p<0.05).

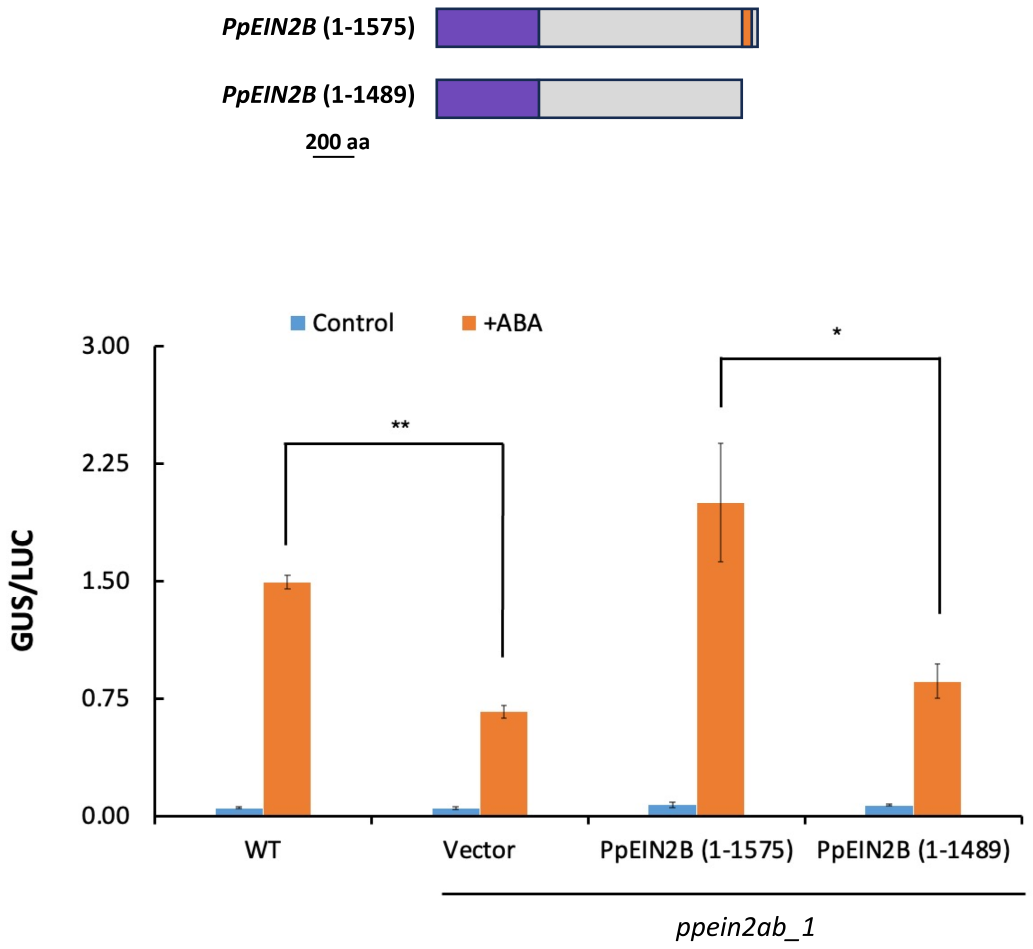

Supplementary Figure S13. Reporter assays with the full-length and C-terminally truncated *PpEIN2B* constructs. The cDNA of *PpEIN2B* with or without the C-terminal deletion of 86 amino acids was introduced into *ppein2ab_1* by particle bombardment to evaluate the abscisic acid (ABA) response recovery. The ABA-inducible *Em* promoter fused with *beta-glucuronidase* (*proEm-GUS*) was used as the reporter, and the rice *Ubiquitin* promoter fused with *luciferase* (*proUbi-LUC*) was used as the reference. After bombardment, the protonemata were cultured for one day with or without 10 μM ABA, and GUS and LUC activity was determined. The values in triplicate were analyzed by Student's t-test. The error bars represent SE (*p<0.05, **p<0.01).

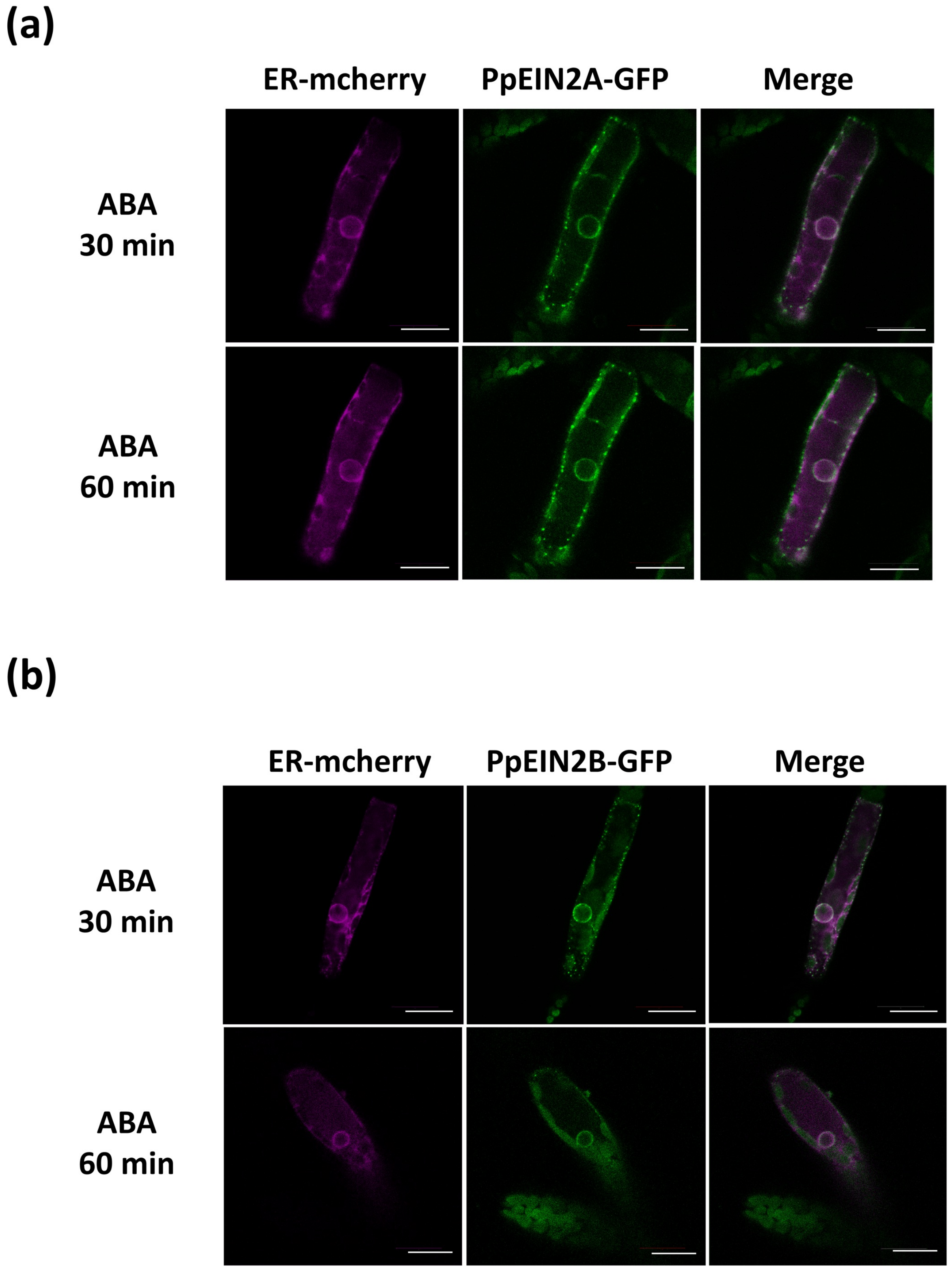

Supplementary Figure S14. Localization of GFP fused to PpEIN2A and PpEIN2B after abscisic acid (ABA) treatment. The protonema cells bombarded with the constructs of the full-length coding regions of *PpEIN2A* (a) and *PpEIN2B* (b) fused to *GFP* were treated with 100 µM ABA for 30 and 60 min and observed under the fluorescent microscope. Red fluorescent protein mCherry with the ER-localization signal (ER-mCherry) was used as a control for the ER-localization. The scale bar indicates 50 µm.

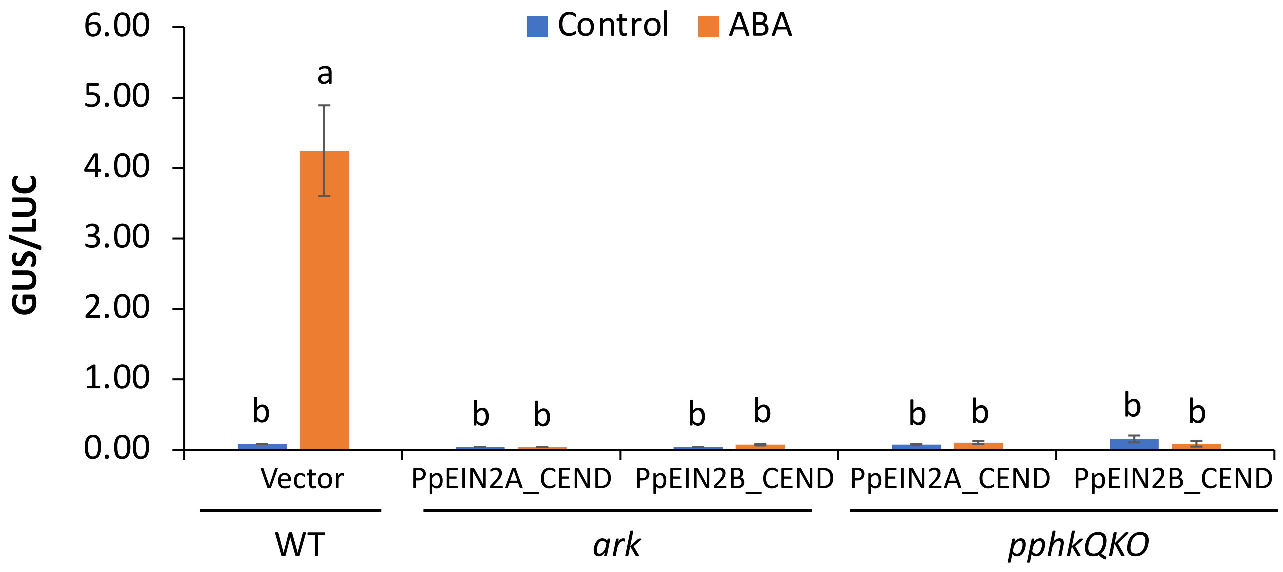

Supplementary Figure S15. Effect of the *PpEIN2_CEND* expression on the abscisic acid (ABA)-induced gene expression in *ark/ctr1l* and *pphkQKO*. Protonemata of wild type (WT), *ark/ctr1l* (*ark*), and *pphkQKO* were cultured for five days and bombarded with the ABA-inducible *Em* promoter fused with *beta-glucuronidase* (*proEm-GUS*) as the reporter, the rice *Ubiquitin* promoter fused with *luciferase* (*proUbi-LUC*) as the reference, and *PpEIN2A_CEND* and *PpEIN2B_CEND* as the effector constructs. After the bombardment, the protonemata were cultured with or without ABA for one day, and the values of GUS and LUC activity were calculated to estimate the ABA response. Standard error (SE) of means (n=3) is indicated. One-way analysis of variance (ANOVA) was performed to compare the means among different groups. Different letters represent the statistical difference (p<0.01).

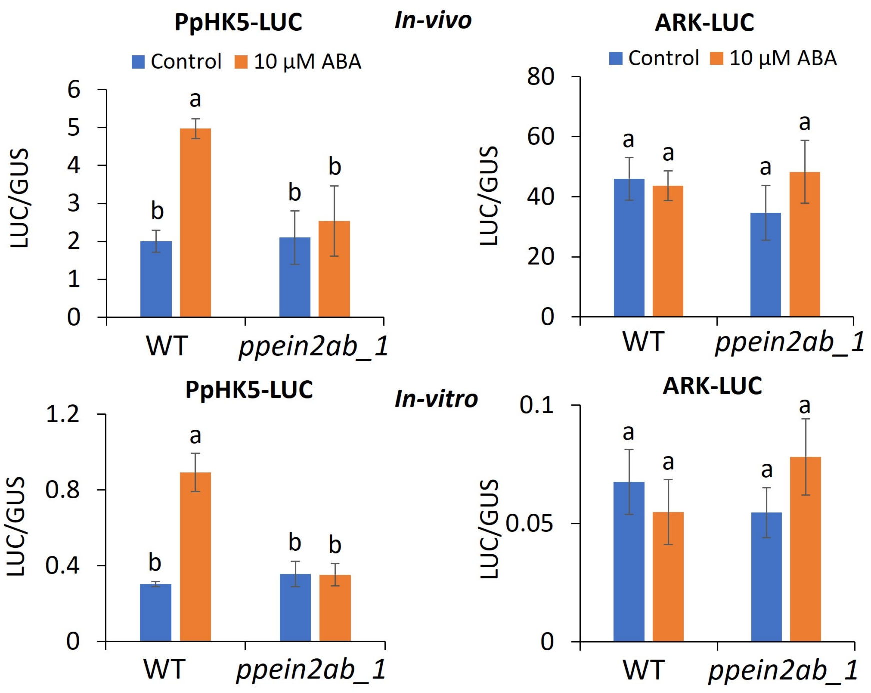

Supplementary Figure S16. Measurement of PpHK5-LUC activity in wild type (WT) and *ppein2ab*. Cultured protonemata were bombarded with *proUbi-PpHK5-LUC* and *proUbi-GUS* constructs. After bombardment, the protonemata were treated with or without 10 µM abscisic acid (ABA) for one day, and activity of LUC relative to GUS was determined both *in vivo* and *in vitro*. Comparison was made using one-way ANOVA. The error bars indicate SE (n=3). Different letters indicate statistical difference (p<0.05).

Supplementary Table S1. Primers used for the RT-qPCR analysis.

Gene names		Nucleotide sequence
Actin	s	5’-AATGAAGTGCGACGTGGATA-3’
	a	5’-CTGACAAGGTGTGATAGCAT-3’
17B9	s	5’-CATACGAGGCTAAAGACGCTGC-3’
	a	5’-CGACGACTGGTGTAAGCCTAATG-3’
6A5	s	5’-GCTGGTGAAGGTGCAAATTATGCC-3’
	a	5’-CCACTTTACTGCTTTGGGGTGAC-3’
LEA173172	s	5’-GGTGGATATGGTGATCACAGGCATC-3’
	a	5’-CGAATCTCCATAACCAGATTTCGGACC-3’
LEA329550	s	5’-CGAAGCTGAGAAGCTTGGTGACA-3’
	a	5’-CTGGGCATCTCTCTTCAAGTCATGC-3’
Tubulin	s	5’-TCTATCTGTCGACTATGGAA-3’
	a	5’-ATGACATGGATACGCGGGTA-3’
PpPIP2;2	s	5’-CGTGACAATCTGCATCTCCATGA-3’
	a	5’-CGGTGTCGATCGACTTTCACG-3’
PpPIP2;3	s	5’-TGAGACGATGCTACGAGGCTT-3’
	a	5’-GGTACGAGGTAGGTCACTACGT-3’

Sense and anti-sense primers are denoted as ‘s’ and ‘a’, respectively.
